# Photosensitive and dual-targeted chromium nanoparticle delivering small interfering RNA YTHDF1 for molecular-targeted immunotherapy in liver cancer

**DOI:** 10.1186/s12951-024-02612-3

**Published:** 2024-06-19

**Authors:** Shang Chen, Yan He, Xin Huang, Yao Shen, Qingshuang Zou, Gun Yang, Li Fu, Quan Liu, Dixian Luo

**Affiliations:** 1 Department of Laboratory Medicine, Huazhong University of Science and Technology Union Shenzhen Hospital (Nanshan Hospital), Shenzhen University, Shenzhen, 518052 People’s Republic of China; 2 Guangdong Key Laboratory for Biomedical Measurements and Ultrasound Imaging, National-Regional Key Technology Engineering Laboratory for Medical Ultrasound, School of Biomedical Engineering, Shenzhen University Medical School, Shenzhen, 518060 People’s Republic of China; 3 Institute of Pharmacy and Pharmacology, School of Pharmaceutical Science, Hengyang Medical School, University of South China, Hengyang, 421001 People’s Republic of China; 4 Department of Thoracic Surgery, The First Affiliated Hospital of Anhui Medical University, Hefei, 230032 People’s Republic of China; 5 Department of Chemistry, The Chinese University of Hong Kong, Shatin, N.T., Hong Kong People’s Republic of China; 6 Guangdong Provincial Key Laboratory of Regional Immunity and Diseases, Department of Pharmacology and International Cancer Center, Shenzhen University Health Science Center, Shenzhen, 518055 People’s Republic of China

**Keywords:** Liver cancer, Tumor-associated macrophage, Dual-targeting, Small interfering RNA, m6A reader YTHDF1

## Abstract

Tumor-associated macrophages (TAMs) are a promising target for cancer immunotherapy, but delivering therapeutic agents to TAMs within the tumor microenvironment (TME) is challenging. In this study, a photosensitive, dual-targeting nanoparticle system (M.RGD@Cr-CTS-siYTHDF1 NPs) was developed. The structure includes a shell of DSPE-modified RGD peptides targeting integrin receptors on tumor cells and carboxymethyl mannose targeting CD206 receptors on macrophages, with a core of chitosan adsorbing m6A reading protein YTHDF1 siRNA and chromium nanoparticles (Cr NPs). The approach is specifically designed to target TAM and cancer cells, utilizing the photothermal effect of Cr NPs to disrupt the TME and deliver siYTHDF1 to TAM. In experiments with tumor-bearing mice, M.RGD@Cr-CTS-siYTHDF1 NPs, when exposed to laser irradiation, effectively killed tumor cells, disrupted the TME, delivered siYTHDF1 to TAMs, silenced the YTHDF1 gene, and shifted the STAT3-STAT1 equilibrium by reducing STAT3 and enhancing STAT1 expression. This reprogramming of TAMs towards an anti-tumor phenotype led to a pro-immunogenic TME state. The strategy also suppressed immunosuppressive IL-10 production, increased expression of immunostimulatory factors (IL-12 and IFN-γ), boosted CD8 + T cell infiltration and M1-type TAMs, and reduced Tregs and M2-type TAMs within the TME. In conclusion, the dual-targeting M.RGD@Cr-CTS-siYTHDF1 NPs, integrating dual-targeting capabilities with photothermal therapy (PTT) and RNA interference, offer a promising approach for molecular targeted cancer immunotherapy with potential for clinical application.

**Graphical Abstract:**

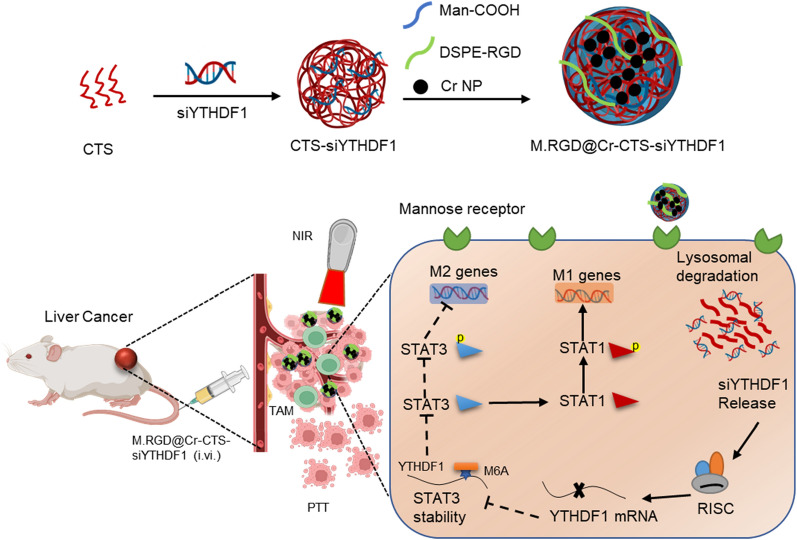

**Supplementary Information:**

The online version contains supplementary material available at 10.1186/s12951-024-02612-3.

## Introduction

Global Cancer Statistics Report 2020 ranks liver cancer as the sixth most diagnosed and third leading cause of cancer-related deaths globally, with approximately 906,000 new cases and 830,000 fatalities [1]. Hepatocellular carcinoma (HCC), a primary liver cancer stemming from chronic tissue damage, is particularly aggressive [2]. Surgical resection is the only curative option, with sorafenib as the sole approved drug for HCC [3]. The 5-year survival rate for liver cancer remains under 30% [4]. Recent research underscores the critical role of RNA epigenetic dysregulation in liver cancer progression, with N6-methyladenosine (m6A) modifications being a key factor. Disruptions in m6A regulatory factors can profoundly affect cancer biology, including cell cycle control, apoptosis resistance, immune evasion, and metastasis [5]. YTHDF1, a central protein in HCC research, has been linked to poor patient outcomes due to its overexpression in liver cancer tissues [6, 7]. Elevated YTHDF1 levels are associated with increased NOTCH1 expression, a tumor stem cell marker, and enhanced HCC stemness, which can lead to drug resistance [8]. In vivo studies show that YTHDF1 siRNA delivered via nanoliposomes significantly suppresses HCC growth and enhances the efficacy of sorafenib and Lenvatinib [8]. Despite the known impact of YTHDF1 on tumor cells, its role in the tumor microenvironment (TME) is less well-documented.

Immunotherapy has advanced in treating cancer, yet solid tumors respond at a rate below 30%, largely due to the immunosuppressive nature of the TME [9, 10]. Tumor-associated macrophages (TAMs), which constitute about 50% of the immune cells within tumors, are pivotal in initiating anti-tumor responses [11]. Studies have revealed the importance of m6A methylation in immune evasion, the absence of the m6A reader YTHDF1 in dendritic cells within the TME improves CD8 + T cell cross-priming and tumor antigen presentation [12]. Similarly, YTHDF1 absence in TAMs promotes anti-tumor immunity in the presence of CD8 + T cells [12]. YTHDF1, an essential m6A reader, selectively binds to methylated RNA to facilitate translation [6]. TAMs transition from M1 to M2 polarization in response to tumor development, a shift that supports tumor growth and immune evasion [13, 14]. However, repolarizing M2-like TAMs to an M1 phenotype can transform the TME into a more immunogenic state [15, 16]. While immune checkpoint inhibitors have shown benefits in advanced HCC, targeting a single pathway may not suffice for effective immunotherapy [17]. Combination strategies targeting multiple immunomodulatory pathways could effectively alter the TME, enhancing anti-tumor immunity and therapeutic outcomes.

Nanoparticles (NPs) offer a promising approach for cancer immunotherapy by delivering drugs to modulate TAM polarization. In our previous research, we found that chromium nanoparticles (Cr NPs) possess both photosensitivity and metal immune properties, which showed improved anticancer effectiveness in hepatoma mouse models by promoting immune cell infiltration and reducing immunosuppression [18]. RNA interference (RNAi), particularly using small interfering RNA (siRNA), has made significant strides in cancer therapy [19]. Given the overexpression of YTHDF1 in TAMs and its role in cancer progression [20], downregulating YTHDF1 in TAMs is expected to reduce M2-type macrophage formation, reshaping the TME for potent antitumor effects.

In this study, we developed photosensitive dual-targeting nanoparticles for delivering short interfering RNAs (siRNAs) that target the m6A reader YTHDF1 to treat hepatocellular carcinoma by modulating epigenetics and the immune response. Our novel photosensitive nanocarrier, M.RGD@Cr-CTS-siYTHDF1 NPs, is composed of chromium nanoparticles (Cr NPs) with siYTHDF1 adsorbed onto chitosan (CTS), encased in a shell of carboxymethyl mannose (Man-COOH, Abbreviations: M.) and decorated by RGD modified with DSPE. This approach disrupts the TME through Cr NPs laser-induced photothermal effects, enabling effective delivery of siYTHDF1 to TAMs to deplete YTHDF1 and inhibit STAT3 protein translation in an m6A-dependent manner, thereby shifting the STAT3-STAT1 equilibrium to enhance STAT1 expression, and achieving improvement of the TME and inhibition of tumor progression. This versatile nanotherapeutic platform offers a highly efficient and low-toxicity method for targeting m6A regulators for siYTHDF1 delivery, highlights the significance of epigenetic modulation in restoring anti-tumor immunity and presents an innovative epigenetic and immune-regulatory approach to cancer therapy.

## Materials and methods

### Materials

High-purity (≥ 99.5%) chromium powder was sourced from Aladdin Co. Ltd. ELISA kits for IFN-γ, TNF-α, IL-10, and IL-12 were purchased from Proteintech Technologies. Shanxi Ruixi Tech Co. Ltd. provided carboxymethyl mannose and DSPE-modified RGD. Rabbit polyclonal antibodies to YTHDF1, STAT1, p-STAT1, STAT3, p-STAT3, NOS2, Arginase-1, CD206, and CD86 were acquired from Abcam Technology. YTHDF1 siRNA (siYTHDF1) and negative control siRNA (siNC) were obtained from Sangon Biotech (Shanghai, China). Primers were procured from Hechengyuan Biotechnology (Shenzhen, China). The primers and sequences for human and mouse YTHDF1 siRNA were detailed in Supplementary Table 1. RAW 264.7 (mouse monocyte macrophage), THP-1 (human monocyte macrophage), and Hepa1-6 (mouse hepatocellular carcinoma) cells were obtained from the Cell Bank of the Shanghai Institutes for Biological Sciences, Chinese Academy of Sciences. Hepa1-6 and RAW 264.7 cells were cultured in DMEM (Gibco, Invitrogen), while THP-1 cells were cultured in RPMI 1640 Medium (Gibco, Invitrogen). Culture media for all cells were supplemented with 10% FBS (Gibco) and 1% penicillin/streptomycin (Gibco) and maintained at 37 °C in a 5% CO_2_ incubator.

### Bioinformatics analysis

Bioinformatics websites, such as GEPIA (http://gepia.cancer-pku.cn), UALCAN (http://ualcan.path.uab.edu/index.html), TIMER 2.0 (http://timer.cistrome.org), and Kaplan–Meier Plotter (https://kmplot.com/analysis/index.php?p=service&cancer=liver_rnaseq), were utilized. Samples of patients with HCC were downloaded from The Cancer Genome Atlas (TCGA, https://www.cancer.gov/tcga/), comprising 270 cases of high YTHDF1 expression and 94 cases of low YTHDF1 expression. These sample data underwent processing using Gene Set Enrichment Analysis software (GSEA v4.0.1) for BIOCARTA, HALLMARK, and Kyoto Encyclopedia of Genes and Genomes (KEGG) analyses as previously described [7, 21].

### Synthesis of M.RGD@Cr-CTS-siYTHDF1 NPs

A 0.02% wt/vol working solution of chitosan (CTS) was prepared by dissolving it in a sodium acetate buffer (0.1 M sodium acetate mixed with 0.1 M acetic acid, pH 4.5). This chitosan solution (100 µl) was then mixed with 32 µg of double-stranded siRNA in a 100 µl 50 mM sodium sulfate solution. The electrostatic interaction between CTS and dsRNA was essential for their stoichiometric balance. The mixture was heated to 55 °C for 2 min and then vortexed at high speed for 1 min to encapsulate the siRNA within NPs. Post-centrifugation at 13,000*g* for 10 min, the particles were vortexed with carboxymethyl mannose (Man-COOH), DSPE-RGD, and Cr NPs at respective concentrations of 1, 1, 0.5, and 0.1 mg/mL for 20 min. The final step involved a 30-min incubation at 37 °C to form the complexes.

### Characterization of M.RGD@Cr-CTS-siYTHDF1 NPs

The size and electrophoretic properties of the resulting NPs were measured using a Nano Zetasizer (Malvern, UK). The morphology of the M.RGD@Cr-CTS-siYTHDF1 NPs was examined under a H7650 Transmission Electron Microscope (TEM, Japan). High-resolution TEM was also employed to assess morphology and elemental composition, along with selected area electron diffraction and energy-dispersive X-ray spectroscopy (EDS) using a FEI Tecnai G2 F30 field-emission TEM at 300 kV. Atomic Force Microscopy (AFM) on a BRUKER Dimension Fastscan was utilized to determine the morphology and height profile of the NPs. Optical absorbance was evaluated by recording UV–visible spectra over the range of 300 nm to 800 nm with a DU-730 spectrophotometer (Beckman, USA) and a HITACHI UH4150 spectrophotometer.

### Cytotoxicity of M.RGD@Cr-CTS-siYTHDF1 in vitro

RAW 264.7, Hepa1-6, and THP-1 cells were enzymatically harvested and seeded into 96-well plates at a density of 10^5^ cells per well (n = 4) overnight. Subsequently, the medium was refreshed with growth medium containing varying concentrations of M.RGD@Cr-CTS-siYTHDF1 (0, 25, 50, and 100 ppm in 100 µL). Cytotoxicity was evaluated after 24 and 48 h of incubation using the Cell Counting Kit-8 (CCK-8, MCE, USA).

### Cell uptake of M.RGD@Cr-CTS-siYTHDF1

For cellular uptake studies, Hepa1-6 cells were seeded into 12-well plates and incubated overnight to facilitate adhesion. The medium was then replaced with Cy5.5-labeled M.RGD@Cr-CTS-siYTHDF1 nanoparticles (100 ppm, 100 µL; n = 4) and incubated for an additional 4 h. Post-incubation, cells were stained with Hoechst 33,342 and Phalloidin (Abcam, UK). The uptake of the labeled nanoparticles was visualized under an Olympus FV3000 inverted fluorescence microscope.

### Isolation and culture of Bone marrow-derived macrophages (BMDMs)

BMDMs were extracted from C57BL/6 (B6) mice with 75% ethanol for 15 min after sacrifice. The harvested cells were filtered through a 70 µm nylon mesh to eliminate debris and then treated with ice-cold red blood cell lysis buffer for 10 min to lyse erythrocytes. Post-lysis, the remaining cells were cultured in Iscove's Modified Dulbecco’s Medium (IMDM, Gibco, Invitrogen), supplemented with 50 ng/mL macrophage colony-stimulating factor (M-CSF, PeproTech, USA). The culture was maintained for 3 days in 10 cm tissue culture plates. On day 5, the medium was refreshed. By day 7, BMDMs were induced for M1 polarization with 100 ng/mL lipopolysaccharide (LPS) or 50 ng/mL interferon-gamma (IFN-γ). For M2 polarization, cells were treated with 10 ng/mL interleukin-4 (IL-4) and/or 10 ng/mL interleukin-13 (IL-13). Antibodies against NOS2 and ARG-1 were used to stain the BMDMs, and Western blotting was conducted to assess their polarization status.

### Cell viability detection by fluorescence imaging

Hepa1-6 cells were cultured with 100 ppm M.RGD@Cr-CTS-siYTHDF1 NPs overnight, and then illuminated with an 808 nm laser (1.0 W/cm^2^, 8 min). After 12 h, cells were incubated with Calcein-AM and PI solution (Beyotime, China) according to the manufacturer’s instructions. Living cells (green fluorescence) and dead cells (red fluorescence) were observed using a fluorescence microscope (Olympus IX71, Japan).

### In vitro photothermal effect of M.RGD@Cr-CTS-siYTHDF1 NPs

Hepa1-6 cells (n = 4) were plated in 96-well plates at a density of 10^5^ cells/well and incubated overnight. The cells were treated with increasing concentrations of M.RGD@Cr-CTS-siYTHDF1 NPs (0, 25, 50, and 100 ppm in 100 μL) in DMEM medium for 8 h. Following treatment, each well was exposed to an 808 nm laser for 8 min at a power density of 1.0 W/cm^2^. Cell viability was evaluated 12 h later using the CCK-8 assay and quantified with a microplate spectrophotometer at 450 nm.

### The toxicity of M.RGD@Cr-CTS-siYTHDF1 NPs in vivo

Female, 6-week-old C57BL/6 mice (16–20 g) were obtained from Beijing Vital River Laboratory Animal Technology Co., Ltd. The mice were maintained under specific pathogen-free conditions with a 12-h light/dark cycle and provided with food and water ad libitum. For toxicity assessment, 24 mice were randomly divided into three groups. Each group received an intravenous injection of either M.RGD@Cr-CTS-siYTHDF1 NPs in phosphate-buffered saline (PBS) at a dosage of 1 mg/kg in 100 µL, or PBS alone as a control. On days 1, 14, and 30 post-injection, blood, serum, and major organs (heart, liver, spleen, lung, and kidney) were harvested for analysis. This included a complete blood count, serum biochemistry to assess liver and kidney function, and histological examination with hematoxylin and eosin (H&E) staining.

### In vivo biodistribution and photothermal effect of M.RGD@Cr-CTS-siYTHDF1 NPs

Hepa1-6 cells were utilized to create subcutaneous hepatic tumors in BALB/c nude mice. When tumor volumes reached 200 mm^3^, mice were randomly assigned to two groups and administered an intravenous injection of either phosphate-buffered saline (PBS) or Cy5.5-labeled M.RGD@Cr-CTS-siYTHDF1 NPs (1 mg/mL, 100 µL, n = 6). Fluorescence imaging was performed using the IVIS Spectrum CT system (MA, USA) at 24, 48, and 72 h post-injection to track the biodistribution. The photothermal effects of the NPs were assessed under 808 nm laser irradiation at 1 W/cm^2^ for 5 min. Temperature changes were recorded at 0, 0.5, 1, 3, and 5 min using a thermometer to determine the heat generation profile induced by laser exposure.

### Reverse transcription-quantitative PCR (RT-qPCR)

Total RNA was extracted from samples using Trizol reagent (Life Technologies, USA) and converted to complementary DNA (cDNA) using the PrimeScript RT Reagent Kit (Vazyme, China) on a 96-Well Thermal Cycler (Applied Biosystems, USA). Quantitative PCR (qPCR) was conducted with the One-Step TB Green PrimeScript RT-PCR Kit II (Vazyme, China) in technical triplicates on a ViiA 7 Real-Time PCR System (Thermo Fisher Scientific, USA), formatted for a 96-well plate. GAPDH was employed as an endogenous reference gene for data normalization. Relative gene expression levels were calculated using using the 2^−ΔΔCT^ method.

### RNA-sequencing

Total RNA was extracted and purified using the RNeasy Mini Kit (QIAGEN, Germany). Sequencing of the RNA libraries was performed on the Illumina HiSeq 4000 platform, adhering to the manufacturer's protocol (Illumina, USA). Gene expression levels were quantified using the Fragments per Kilobase of transcript per Million mapped reads (FPKM) method with the DESeq2 software package. Pathway analysis was conducted via Over-Representation Analysis (ORA) utilizing the KEGG database.

### MeRIP-qPCR

Total RNA was isolated using Trizol reagent (Life Technologies), and any potential DNA contamination was removed using DNase from the PrimeScript RT Reagent Kit (Vazyme, China). RNA fragmentation was achieved with RNA Fragmentation Buffer (10 mM Tris–HCl, 10 mM ZnCl2) at 70 °C for 5 min. Fragmented RNA was incubated with Protein A/G Magnetic Beads (ThermoFisher, USA) pre-bound to an anti-m6A antibody (Abcam, UK) at 4 °C for 4 h in the presence of an RNase inhibitor. The beads were sequentially washed with IP buffer, low-salt IP buffer, and high-salt IP buffer. RNA was eluted using the RLT buffer from the RNeasy Mini Kit (QIAGEN, Germany) and further purified with the DireCTzol RNA Miniprep Kit (Zymo Research, USA). The purified RNA was then subjected to RT-qPCR as per standard laboratory protocols.

### RIP-qPCR

RNA immunoprecipitation (RIP) was conducted with an anti-YTHDF1 antibody using the Magna RIP™ Kit (Merck, Germany), following the provider's guidelines. Cell pellets were lysed in RIP Lysis Buffer and incubated with magnetic beads conjugated to the anti-YTHDF1 antibody overnight at 4 °C. The beads were extensively washed with RIP Wash Buffer (six washes), and the RNA was eluted by digesting the antibody with proteinase K in the presence of 1% (w/v) sodium dodecyl sulfate (SDS) at 55 °C for 30 min. The RNA was then purified through chloroform and ethanol precipitation, resuspended, and quantified via RT-qPCR using established protocols.

### Enzyme-linked immunoassay (ELISA)

H22 tumor samples were collected, weighed, and homogenized in phosphate-buffered saline (PBS) with a protease inhibitor cocktail on ice. Following centrifugation at 12,000 rpm for 5 min at 4 °C, the supernatants were isolated for the detection of IL-10, IL-12, TNFα, and IFN-γ using ELISA kits from Proteintech (USA), in accordance with the manufacturer’s protocol.

### Anticancer evaluation in vivo

Healthy female C57BL/6 mice (5–6 weeks old, weighing 16–20 g) with subcutaneous H22 hepatoma tumor xenografts were used in this study. Tumors were established by injecting 5 × 10^5^ H22 cells into the mice's right flank, targeting a volume of approximately 100 mm^3^. The mice (n = 6) were randomly assigned to one of seven groups: (1) Saline, (2) M.RGD@Cr-CTS-siNC, (3) M.RGD@Cr-CTS-siNC with near-infrared (NIR) laser treatment, (4) M@Cr-CTS-siYTHDF1, (5) M@Cr-CTS-siYTHDF1 with NIR, (6) M.RGD@Cr-CTS-siYTHDF1, and (7) M.RGD@Cr-CTS-siYTHDF1 with NIR. Treatments (5 mg/mL, 100 µL) were administered intravenously on days 2 and 8. Mice in the laser treatment groups were exposed to an 808 nm laser (1 W/cm^2^, 8 min) 24 h after injection. Tumor dimensions and body weight were measured every 2 days with digital calipers. Tumor volume was calculated using the formula V = (length × width^2^)/2. After 18 days, mice were euthanized, and tumors were excised, fixed in 4% formalin, paraffin-embedded, and subjected to immunohistochemical staining for YTHDF1 and immunofluorescence staining for Ki67, CD25, CD86, CD206, CD4, CD8, and H&E. ELISA was also used to measure IL-10 and IFN-γ in tumor homogenates and IL-12 and IL-1β in mouse serum.

### Statistical analysis

Statistical analysis utilized SPSS 26.0 software (SPSS, Inc., Chicago, USA). Data were analyzed through one-way ANOVA with post hoc LSD analysis or unpaired two-tailed Student’s t-test. Results were presented as mean ± S.D. Statistical significance was defined as p < 0.05.

## Results and discussion

### High YTHDF1 expression in liver cancer and M2-type macrophages correlates with poor prognosis

Analysis of YTHDF1 expression in hepatocellular tumor samples from The Cancer Genome Atlas (TCGA) and Genotype-Tissue Expression (GTEx) databases using RNA sequencing (RNA-seq) showed significant upregulation in liver cancer tissues (Supplementary Fig. 1A), which was associated with worse overall survival (Supplementary Fig. 1B). These findings have been consistently documented in multiple studies [6–8]. Consistent overexpression of YTHDF1 in liver cancer tissues was confirmed through immunohistochemical and qPCR analyses of 23 pairs of liver cancer samples (Supplementary Fig. 1C, D). TIMER 2.0 database analysis revealed a positive correlation between M2-type macrophage infiltration and YTHDF1 expression in liver cancer (Supplementary Fig. 1E). Additionally, YTHDF1 expression was positively correlated with myeloid-related genes REL and FUT4 (Supplementary Fig. 1F), and high infiltration of M2-type macrophages expressing YTHDF1 was linked to a significantly poorer prognosis (Supplementary Fig. 1G). Immunofluorescence staining of 10 liver cancer tissue samples showed a notable increase in co-localization of M2-type macrophages (CD206, green) and YTHDF1 (red) compared to normal liver tissues (Supplementary Fig. 1H, I). These results suggest that YTHDF1 upregulation in liver cancer cells and M2-type tumor-associated macrophages influences patient prognosis.

### Preparation and physicochemical characterization of M.RGD@Cr-CTS-siYTHDF1 NPs

**Fig. 1:**
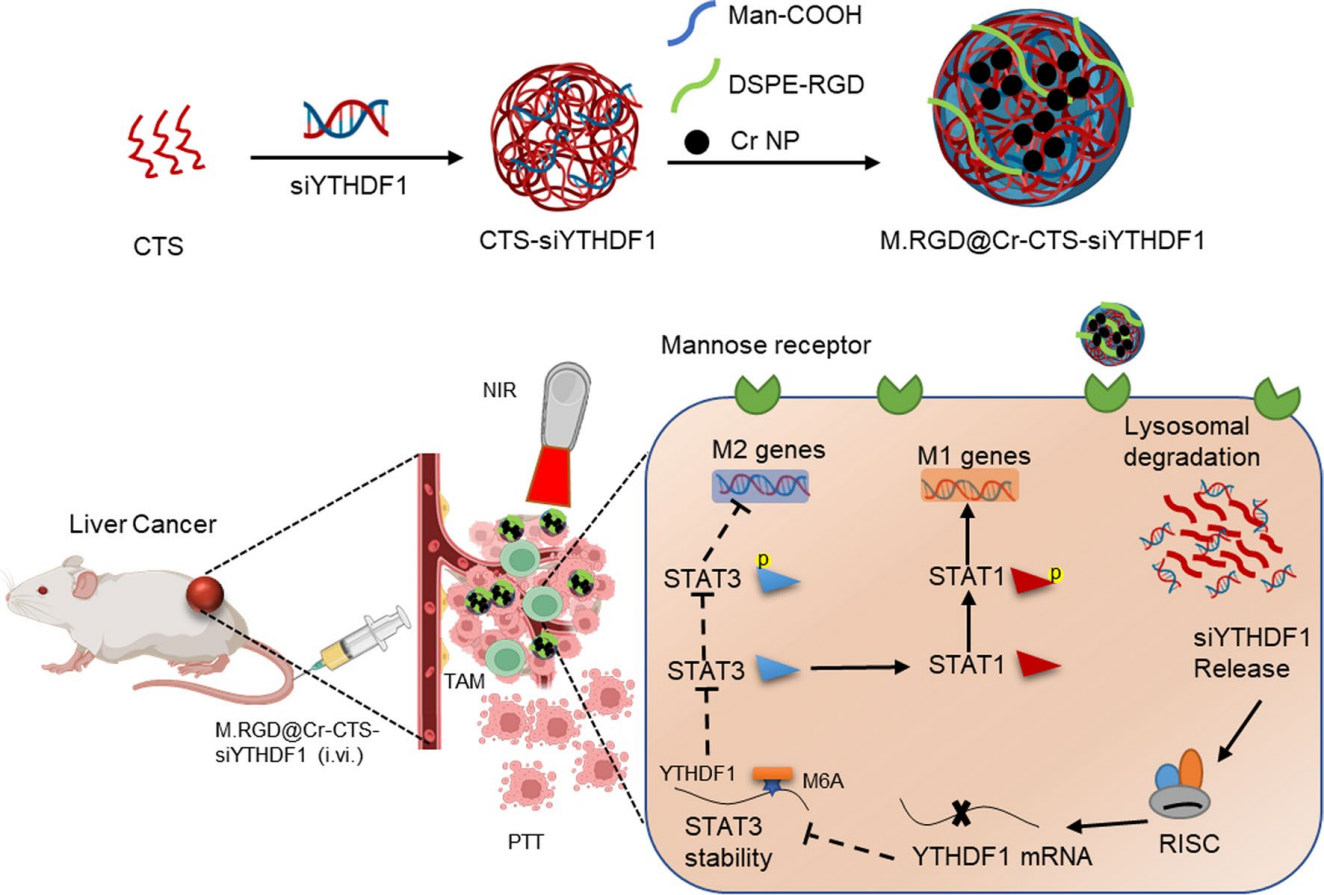
Design of the M.RGD@Cr-CTS-siYTHDF1 NPs for TAM and tumor specific molecular-targeted and synergistic photothermal therapy (PTT)

M.RGD@Cr-CTS-siYTHDF1 NPs were synthesized by utilizing a core comprising chromium nanoparticles (Cr NPs) and CTS-siYTHDF1, along with an outer shell of carboxymethyl mannose (Man-COOH, abbreviated as M.), which was decorated with DSPE-RGD (Fig. 1). The self-assembly of polycations with dsRNA, facilitated by the electrostatic forces between the positive charges of the amino group in chitosan and the negative charges carried by the phosphate groups on the dsRNA backbone, was utilized to form chitosan/dsRNA (CTS-siRNA) complexes [22]. Man-COOH has often been employed as a nanoparticle shell in various studies [23, 24]. In this study, Man-COOH was used as the encapsulation material to enclose the CTS-siRNA complexes and Cr NPs through vortexing and incubation processes. Further vortexing and incubation, followed by the addition of DSPE-RGD, led to the formation of M.RGD@Cr-CTS-siYTHDF1 NPs. The hydroxyl groups present in Man-COOH can form hydrogen bonds with the amine, hydroxyl, or carbonyl groups in DSPE-RGD, which contribute to the formation and stabilization of the complex [25, 26]. Additionally, the negatively charged carboxyl groups of Man-COOH can interact electrostatically with the positively charged regions of DSPE-RGD, thus stabilizing the complex and enhancing the overall formulation integrity [27, 28].

**Fig. 2:**
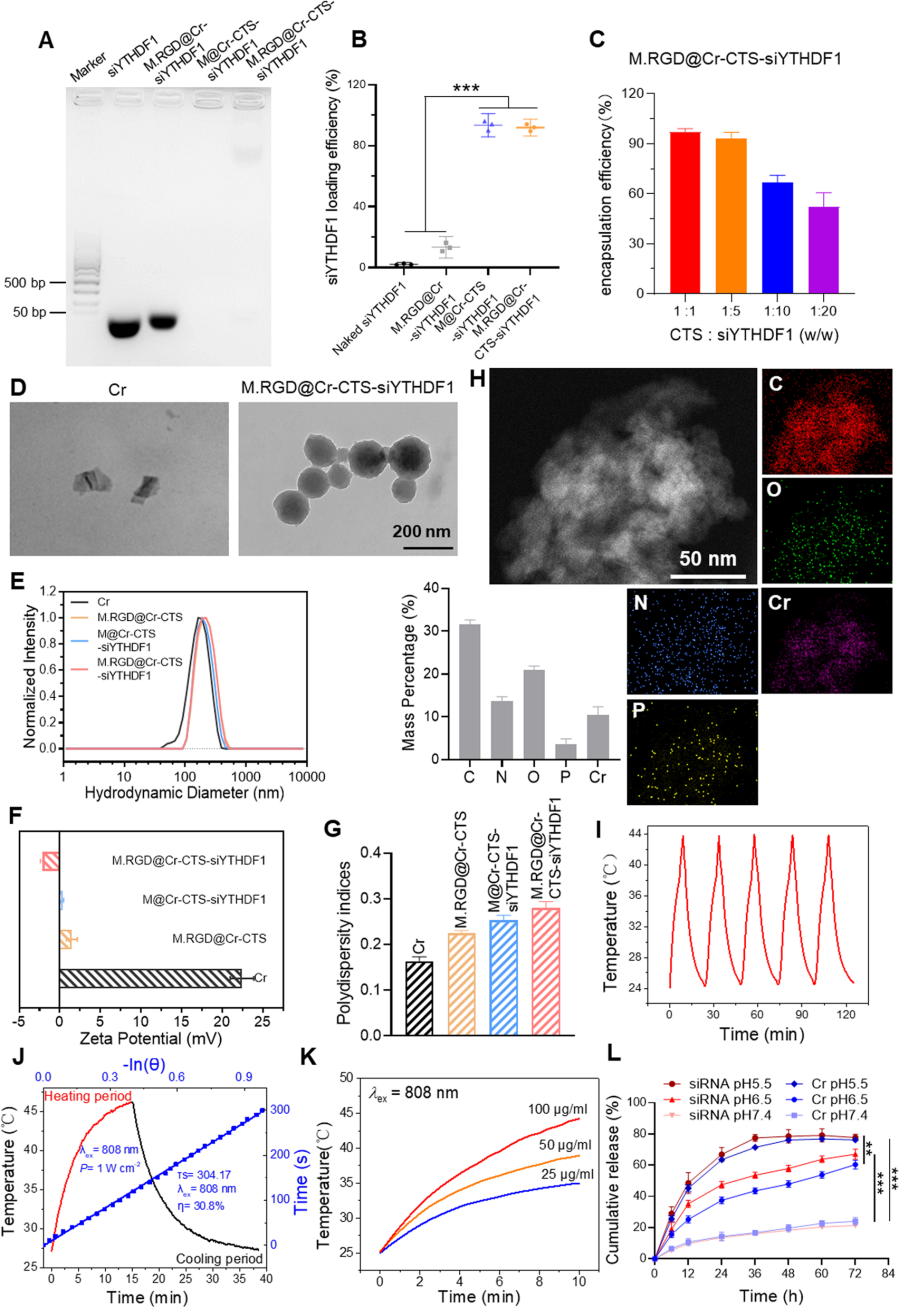
Preparation and characterization of M.RGD@Cr-CTS-siYTHDF1 NPs. **A** Agarose gel electrophoresis assessment of dsRNA retention in NPs including naked siRNA, M.RGD@Cr-siYTHDF1, M.@Cr-CTS-siYTHDF1, and M.RGD@Cr-CTS-siYTHDF1 after 24-h storage in PBS. **B** Detection of RNA release content in the supernatant after 24-h storage of naked siRNA, M.RGD@Cr-siYTHDF1, M.@Cr-CTS-siYTHDF1, and M.RGD@Cr-CTS-siYTHDF1 in PBS. Data are presented as the means ± SD (n = 3). **C** Encapsulation efficiency of siRNA in M.RGD@Cr-CTS-siYTHDF1 at different mass ratios of siRNA to chitosan. **D** Transmission electron microscopy (TEM) and energy dispersive X-ray spectroscopy mapping of M.RGD@Cr-CTS-siYTHDF1 NPs (scale bar = 200 nm). **E** DLS size distribution profile of four types of NPS (Cr, M.RGD@Cr-CTS, M@Cr-CTS-siYTHDF1, and M.RGD@Cr-CTS-siYTHDF1). **F** Zeta potential measurements of Cr NP, M.RGD@Cr-siYTHDF1, M.@Cr-CTS-siYTHDF1, and M.RGD@Cr-CTS-siYTHDF1. **G** Polydispersity index (PDI) values of the four types of NPs (Cr, M.RGD@Cr-CTS, M@Cr-CTS-siYTHDF1, and M.RGD@Cr-CTS-siYTHDF1) in PBS. **H** The energy-dispersive X-ray spectroscopy mapping images of M.RGD@Cr-CTS-siYTHDF1 NPs and element percentages of elements C, P, O, N and Cr in M.RGD@Cr-CTS-siYTHDF1 NPs. Scale bars, 50 nm. **I** Heating curves of M.RGD@Cr-CTS-siYTHDF1 suspensions in water for 5 lasers on/off cycles (1 W/cm^2^) under the laser irradiation at 808 nm. **J** Photothermal conversion efficiency in M.RGD@Cr-CTS-siYTHDF1 NPs. **K** Heating curves of different concentrations of M.RGD@Cr-CTS-siYTHDF1 dispersions irradiated by 808 nm laser (1 W/cm^2^). **L** Cumulative release rate of Cr NP and siRNA from M.RGD@Cr-CTS-siYTHDF1 NPs at different pH values (5.5, 6.5 and 7.4). Data are presented as the means ± SD (n = 3), ***p < 0.001

To evaluate the stability and encapsulation efficiency of siRNA within NPs, we prepared three formulations: naked siYTHDF1, CTS-siYTHDF1, and Cr NPs encapsulated with or without RGD modification (M.RGD@Cr-siYTHDF1, M.@Cr-CTS-siYTHDF1, and M.RGD@Cr-CTS-siYTHDF1). Agarose gel electrophoresis was conducted to assess siRNA mobility. As depicted in Fig. 2A, the migration of siRNA in M.@Cr-CTS-siYTHDF1 and M.RGD@Cr-CTS-siYTHDF1 NPs was completely inhibited, suggesting efficient siRNA encapsulation. The supernatant from high-speed centrifugation of the NP solution was analyzed for siRNA loading efficiency. Over 90% of siRNA was retained in M.@Cr-CTS-siYTHDF1 and M.RGD@Cr-CTS-siYTHDF1 NPs, contrasting with naked siRNA and M.RGD@Cr-siYTHDF1 (Fig. 2B). Varying CTS/siRNA weight ratios (w/w) were tested for M.RGD@Cr-CTS-siYTHDF1 NP preparation. Figure 2C shows that when the ratio reached or exceeded 1:5, siRNA binding to CTS plateaued, with encapsulation efficiency exceeding 93%, indicating optimal siRNA loading. A 1:5 CTS/siRNA weight ratio was selected for stable and efficient siRNA delivery. Transmission electron microscopy (TEM) imaging confirmed the spherical morphology of Cr NPs and M.RGD@Cr-CTS-siYTHDF1 NPs, with a mean particle size of approximately 200 nm as measured by DLS (Fig. 2D, E). Zeta potential measurements for Cr, M.RGD@Cr-CTS, and M@Cr-CTS-siYTHDF1 were 24.6 ± 1.8, 2.2 ± 1.1, and 0.7 ± 0.5 mV, respectively. Following siYTHDF1 and RGD incorporation, the zeta potential of M.RGD@Cr-CTS-siYTHDF1 decreased to − 3.2 ± 0.9 mV, indicating successful encapsulation and a charge shielding effect that enhances NP stability in circulation (Fig. 2F). Additionally, the polydispersity index (PDI) of the nanoconstructs was evaluated, with Cr and M.RGD@Cr-CTS exhibiting low PDIs of 0.16 ± 0.04 and 0.22 ± 0.02, respectively, indicating a monodisperse population (Fig. 2G). M@Cr-CTS-siYTHDF1 and M.RGD@Cr-CTS-siYTHDF1, when coupled with siRNA, demonstrated higher PDIs of 0.25 ± 0.03 and 0.27 ± 0.05 (p = 0.1850), respectively. The presence and percentage content of each element in M.RGD@Cr-CTS-siYTHDF1 NPs were determined by energy-dispersive X-ray spectroscopy (EDS), as shown in Fig. 2E. The analysis revealed the co-localization of chromium (Cr), carbon (C), oxygen (O), nitrogen (N), and phosphorus (P) in M.RGD@Cr-CTS-siYTHDF1 NPs, indicating that siRNA and Cr NPs are components of M.RGD@Cr-CTS-siYTHDF1 NPs. The composite material of all formulations of M.RGD@Cr-CTS-siYTHDF1 NPs is primarily composed of C, O, N, P, and Cr, with phosphorus accounting for approximately 3.6% and chromium for 10.5% (Fig. 2H). M.RGD@Cr-CTS-siYTHDF1 NPs exhibited consistent photothermal effects across heating cycles (Fig. 2I), with a photothermal conversion efficiency of 30.8% at 808 nm, aligning with previous research (Fig. 2J). The light-induced release of Cr NPs from the M.RGD@Cr-CTS-siYTHDF1 complex was evaluated under physiological conditions with 808 nm laser irradiation, resulting in a temperature increase from 25 °C to 45 °C within 10 min at a concentration of 100 μg mL^−1^ (Fig. 2K). The UV absorbance intensity of the NPs under near-infrared (NIR) laser irradiation is shown in Supplementary Fig. 2. M.RGD@Cr-CTS-siYTHDF1 NPs were assessed for the release of Cr NPs and siRNA over time in PBS at different pH values (Fig. 2L). Minimal release was observed at near-neutral pH 7.4 (simulated blood flow), while a significantly cumulative release of Cr NPs and siRNA was observed within 72 h at more acidic pH 5.5 (simulated lysosomes) or pH 6.5 (simulated tumor microenvironment). This suggests that the nanoparticles exhibit pH-sensitive characteristics, providing protection to Cr NPs and siRNA within their nanostructure until they encounter the slightly acidic environment of the tumor microenvironment and the lysosome following cellular internalization, triggering its degradation and release of Cr NPs and siRNA. Consistent with previous studies, our M.RGD@Cr-CTS-siYTHDF1 NPs demonstrated high efficacy in delivering photosensitizers and siRNA, potentially enhancing their antitumor effects [29].

### The biocompatibility, photothermal capabilities, and targeting performance of M.RGD@Cr-CTS-siYTHDF1 NPs

**Fig. 3:**
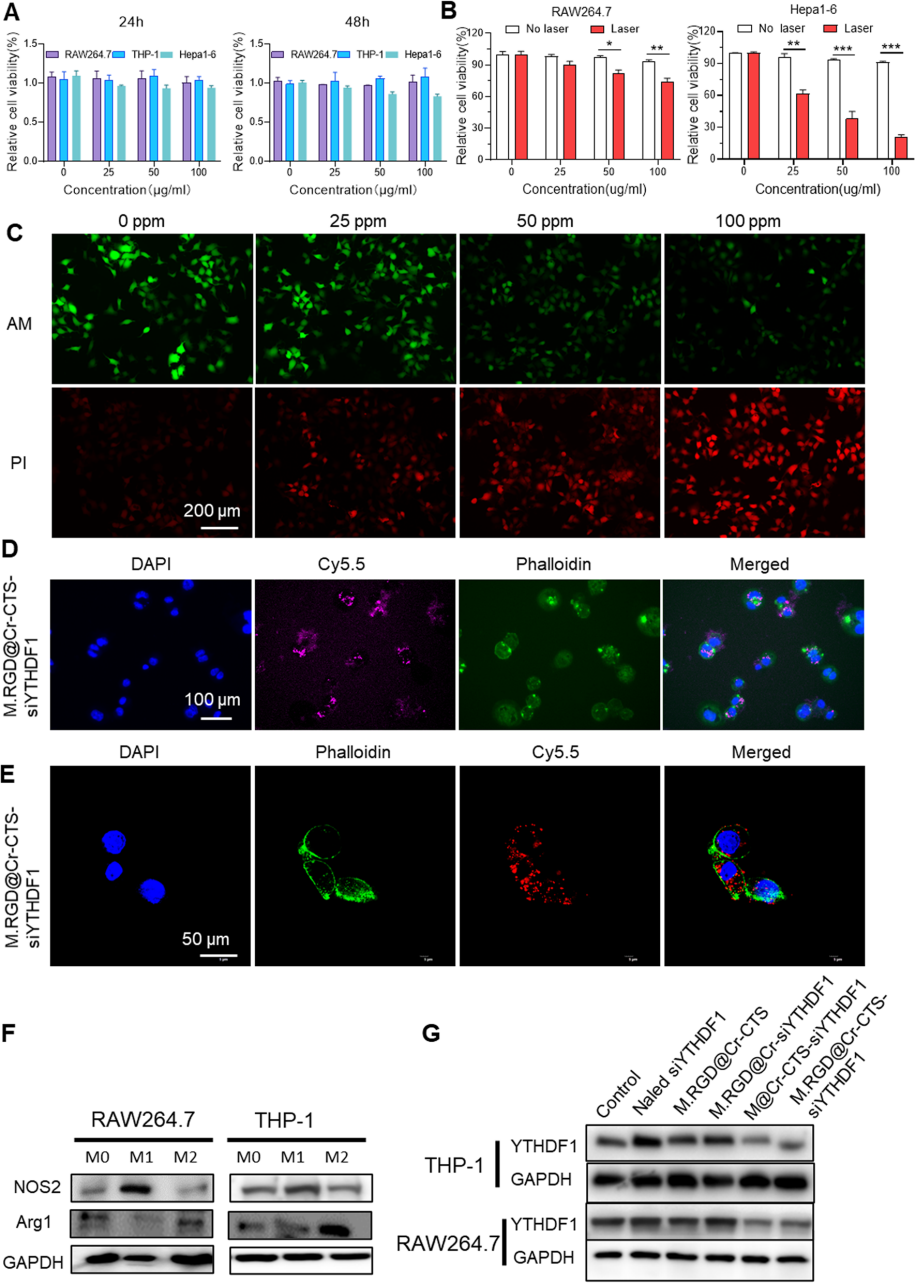
Cytotoxicity, photothermal ablation, and gene knockdown efficiency of M.RGD@Cr-CTS-siYTHDF1 NPs. **A** Cytotoxicity assays of M.RGD@Cr-CTS-siYTHDF1 NPs on Hepa1-6, RAW264.7, and THP-1 cells. **B** Cell viability assessment of photothermal ablation by M.RGD@Cr-CTS-siYTHDF1 NPs on RAW264.7 and Hepa1-6 cells at various concentrations. Data are presented as the means ± SD (n = 3), *p < 0.05, **p < 0.01, ***p < 0.001. **C** Viability of Hepa1-6 cells post-photothermal ablation with different concentrations of M.RGD@Cr-CTS-siYTHDF1 NPs. Scale bar = 100 μm. **D**, **E** Confocal microscopy visualization of the uptake of Cy5.5-labeled M.RGD@Cr-CTS-siYTHDF1 NPs by Hepa1-6 (**D**) and RAW264.7 cells (**E**). **F** Validation of M1 (NOS2) and M2 (Arg1) macrophage polarization using Assessment of YTHDF1 gene knockdown efficiency by M.RGD@Cr-CTS-siYTHDF1 NPs in RAW264.7 and THP-1 cells using Western blotting. **G** Assessment of YTHDF1 gene knockdown efficiency by M.RGD@Cr-CTS-siYTHDF1 NPs in RAW264.7 and THP-1 cells using Western blotting

To assess the in vitro cytotoxicity and biocompatibility of M.RGD@Cr-CTS-siYTHDF1 NPs, we exposed Hepa1-6, RAW264.7, and THP-1 cells to increasing concentrations of the NPs (0, 25, 50, and 100 mg/L) for 24 and 48 h. After 24 h, the NPs showed no cytotoxic effects at any of the tested concentrations (Fig. 3A). Even after 48 h and at a concentration of 100 ppm, the NPs did not exhibit cytotoxic effects in RAW264.7 and THP-1 cells. However, there was a slight decrease in cell viability (85.3%) observed in Hepa1-6 cells after 48 h (Fig. 3A). This may be attributed to the crucial role of the YTHDF1 gene in the growth of liver cancer cells. The uptake of M.RGD@Cr-CTS-siYTHDF1 NPs by Hepa1-6 cells resulted in the knockdown of the YTHDF1 gene, which affected their growth, consistent with previous reports [7, 8]. Therefore, we selected a concentration of 100 ppm for subsequent experiments involving M.RGD@Cr-CTS-siYTHDF1 NPs. A dose-dependent photothermal response was observed in Hepa1-6 cells, which was more pronounced than in RAW264.7 cells, likely due to the RGD peptide in the NPs (Fig. 3B). After a 4-h treatment with M.RGD@Cr-CTS-siYTHDF1 NPs, Hepa1-6 cells showed concentration-dependent apoptosis (indicated by red fluorescence) as a result of localized PTT (Fig. 3C). We assessed the intracellular delivery of M.RGD@Cr-CTS-siYTHDF1 NPs in Hepa1-6 and RAW264.7 cells at different concentrations (0, 25, 50, and 100 μg/ml). After a 4-h treatment with M.RGD@Cr-CTS-siYTHDF1 NPs, we observed their intracellular delivery and localization using fluorescence imaging. The images revealed a dose-dependent uptake of Cy5.5-labeled M.RGD@Cr-CTS-siYTHDF1 NPs by Hepa1-6 and RAW264.7 cells through endocytosis as the concentration increased (Fig. 3D, E, Supplementary Fig. 5A, B). Compared to the control group treated with Cy5.5-CTS-siRNA, the enhanced endocytic uptake of Cy5.5-labeled NPs confirmed more efficient intracellular delivery in both cell types.

To assess the functionality of M.RGD@Cr-CTS-siYTHDF1 NPs, the knockdown effect on YTHDF1 was studied in RAW264.7 and THP-1 cells. M1 and M2 macrophage phenotypes were induced using cytokines (Fig. 3F). YTHDF1 protein knockdown in M2 macrophages derived from RAW264.7 and THP-1 cells were evaluated following treatment with various formulations. Western blot analysis showed a significant reduction in YTHDF1 protein expression in M2-induced macrophages treated with M@Cr-CTS-siYTHDF1 and M.RGD@Cr-CTS-siYTHDF1 NPs (Fig. 3G). These results highlight the NPs’ biocompatibility, photothermal efficacy, and targeting capabilities in reducing YTHDF1 protein levels.

An in-depth toxicology study was conducted using C57BL/6 female mice. Mice were randomly assigned to a control group receiving intravenous saline or an experimental group receiving M.RGD@Cr-CTS-siYTHDF1 NPs (5 mg/kg). On days 1, 14, and 30 post-injection, blood routine, liver and kidney function tests, and histological examination of major organs (lung, liver, spleen, kidney, and heart) were performed. Hematological parameters, including red and white blood cell counts, hematocrit, hemoglobin levels, and platelet measurements, were assessed. No significant hematological toxicity or changes were observed in the NP-treated group at any time point (Supplementary Fig. 3). Liver and kidney functions, evaluated through blood biochemical parameters (albumin, alanine transaminase, aspartate transaminase, creatinine, and blood urea nitrogen), showed no significant differences between the groups (Supplementary Fig. 3). Histological examination with H&E staining revealed no signs of acute or chronic toxicity in major organs on days 1, 14, and 30 (Supplementary Fig. 4). These findings indicate the excellent biocompatibility of M.RGD@Cr-CTS-siYTHDF1 NPs, warranting further investigation for in vivo cancer treatment applications.

### In vivo tumor targeting and distribution of M.RGD@Cr-CTS-siYTHDF1 NPs

**Fig. 4:**
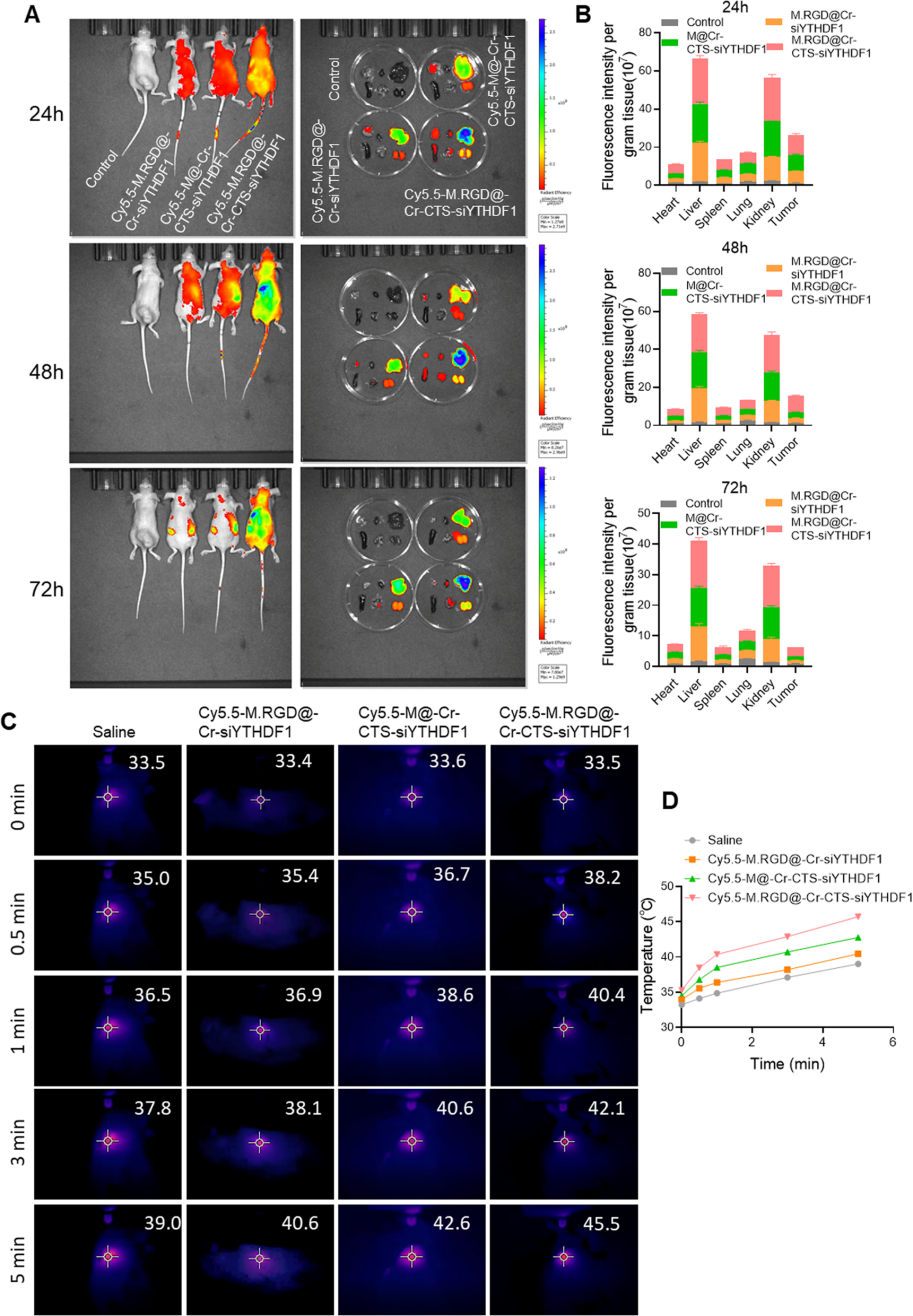
In vivo tumor targeting and distribution of Cy5.5-labeled M.RGD@Cr-CTS-siYTHDF1 NPs. **A** Fluorescence images of the tumor and organs at 24 h, 48 h and 72 h after injecting Cy5.5-labeled NPs (including M.RGD@Cr-CTS, M@Cr-CTS-siRNAs and M.RGD@Cr-CTS-siRNAs). **B** Quantitative distribution analysis of Cy5.5-labeled NPs in nude mice based on the average fluorescence intensity per gram in tumors and organs. **C** Thermal maps and the increase in temperature over time in H22 tumor-bearing mice exposed to an 808 nm laser (1 W cm ^−2^, 8 min) and injected with 100 μL of saline, M.RGD@Cr-CTS NPs (1 mg mL ^−1^), M@Cr-CTS-siRNAs NPs (1 mg mL ^−1^) and M.RGD@Cr-CTS-siRNAs (1 mg mL ^−1^). **D** Temporal temperature changes in H22 tumor-bearing mice following different treatments in **C**

Cy5.5-labeled M.RGD@Cr-siYTHDF1, M.@Cr-CTS-siYTHDF1, and M.RGD@Cr-CTS-siYTHDF1 NPs were administered intravenously to BALB/c nude mice bearing Hepa1-6 tumors for biodistribution assessment using fluorescence imaging. Ex vivo fluorescence imaging of the tumor, lungs, kidneys, heart, liver, and spleen was performed at 24 and 48 h post-injection. The group without chitosan for siRNA adsorption (M.RGD@Cr-siYTHDF1) displayed rapid metabolism and significant NP accumulation in the tumor tissue Fig. 4A, B). By 72 h post-injection, M.RGD@Cr-CTS-siYTHDF1 NPs demonstrated enhanced tumor accumulation compared to M@Cr-CTS-siYTHDF1, suggesting a prolonged residence in the tumor microenvironment, which is beneficial for therapeutic efficacy.

The photothermal effects of M.RGD@Cr-siYTHDF1, M.@Cr-CTS-siYTHDF1, and M.RGD@Cr-CTS-siYTHDF1 NPs with active tumor targeting were assessed using a photothermal imaging system. Figure 3C illustrates the effective photothermal response, which was dependent on the duration of near-infrared (NIR) exposure. Primary tumor temperatures in the M.RGD@Cr-siYTHDF1 and M.@Cr-CTS-siYTHDF1 groups increased to over 40 °C from approximately 33.5 °C during a 5-min irradiation period. Notably, M.RGD@Cr-CTS-siYTHDF1 NPs achieved a temperature of 45.5 °C, surpassing the other groups (40.6 and 42.6 °C), effectively inducing tumor cell death. This contrasts with the minimal temperature change observed in the Saline group (Fig. 4C, D). These results highlight the superior synergistic photothermal effect of M.RGD@Cr-CTS-siYTHDF1 NPs in vivo, which promotes immunogenic cell death (ICD) and tumor cell rupture. Collectively, these findings emphasize the exceptional biocompatibility, photothermal efficacy, and tumor-targeting capabilities of M.RGD@Cr-CTS-siYTHDF1 NPs for cancer cell ablation upon laser triggering.

### The impact of YTHDF1 depletion on macrophage

**Fig. 5:**
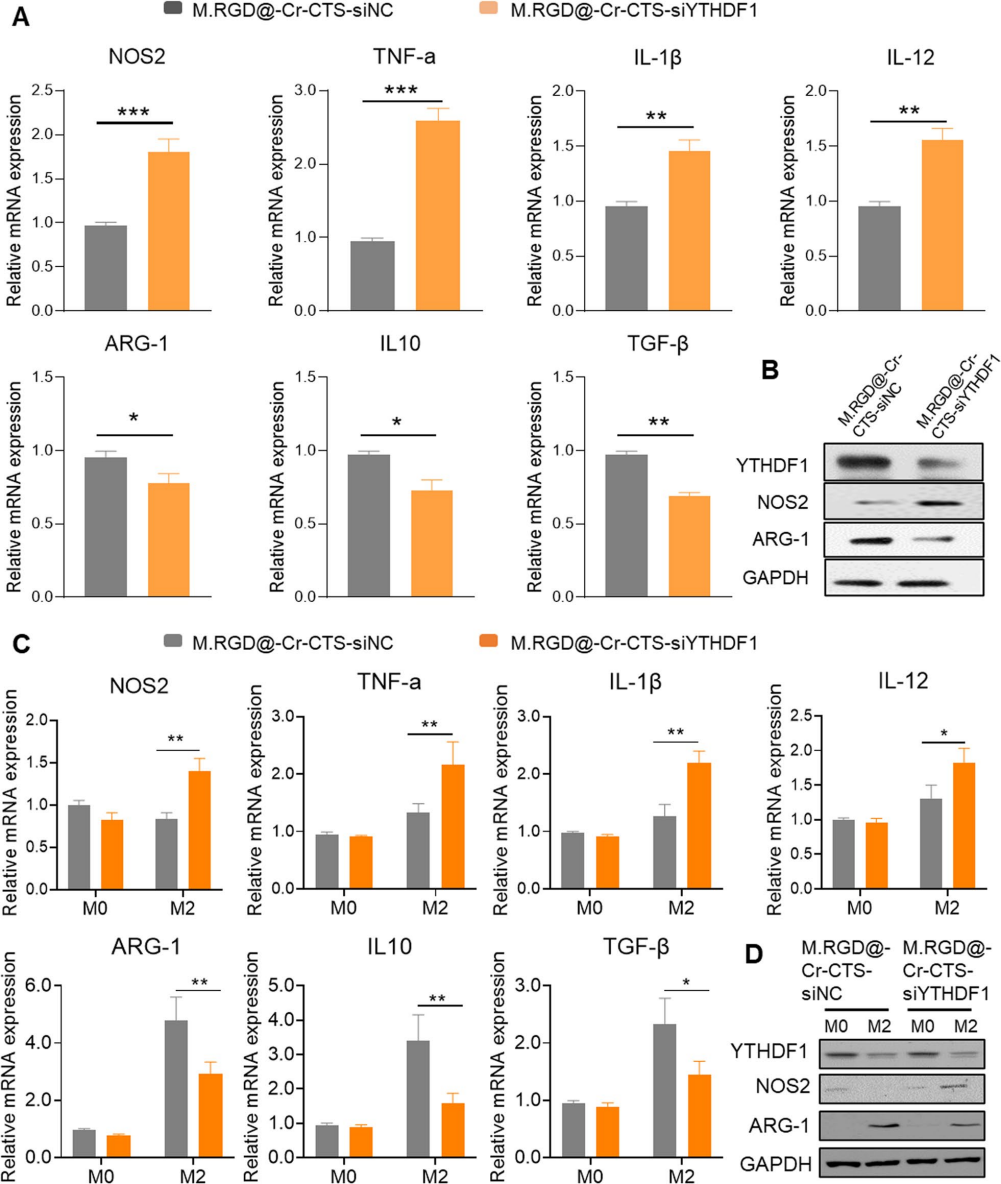
M.RGD@Cr-CTS-siYTHDF1 NPs enhance M1 phenotype by depleting YTHDF1 in M2-type macrophages. **A** qPCR analysis of M1 markers TNF-α, IL-1β, IL-12, NOS2, and M2 markers IL10, ARG-1, TGF-β mRNA expression in THP-1 derived M2- type cells. **B** Western blot analysis for the expression of YTHDF1, NOS2, ARG-1, and GAPDH proteins in M.RGD@Cr-CTS-siNC and M.RGD@Cr-CTS-siYTHDF1 NPs treated THP-1 cells. **C** qPCR analysis of M1 markers Nos2, Tnf-α, Il-1β, Il-12, and M2 markers Il10, Arg-1, Tnf-β mRNA expression in M.RGD@Cr-CTS-siNC and M.RGD@Cr-CTS-siYTHDF1 NPs treated BMDMs-derived M0 and M2-type macrophages. **D** Western blot analysis of the expression levels of Ythdf1, Nos2, Arg-1, and Gapdh proteins. Data are presented as the means ± SD (n = 3), *p < 0.05, **p < 0.01, ***p < 0.001

In THP-1 cells, M.RGD@Cr-CTS-siYTHDF1 NPs were used for a 48-h co-incubation, followed by the addition of 20 ng/ml IL-4 and 20 ng/ml IL-13 to induce M2-type macrophage differentiation. Compared to the M.RGD@Cr-CTS-siNC NPs group, the expression of M1-type genes (NOS2, TNF-α, IL-1β, and IL-12) was significantly upregulated, whereas M2-type genes (ARG-1, IL10, and TGF-β) showed a downward trend (Fig. 5A). At the protein level, YTHDF1 knockdown by M.RGD@Cr-CTS-siYTHDF1 NPs in THP-1 cells was accompanied by a notable reduction in the M2-type macrophage marker ARG-1 and a significant increase in the M1-type marker NOS2 (Fig. 5B).

To further investigate the effect of YTHDF1 absence in bone marrow-derived macrophages (BMDMs), M.RGD@Cr-CTS-siYTHDF1 NPs were added after a 48-h incubation period, followed by the addition of 20 ng/ml IL-4 to promote M2-type macrophage differentiation. In M0 BMDMs, YTHDF1 depletion did not alter the expression of M1 and M2-type genes. However, in M2-type BMDMs, the absence of YTHDF1 led to a significant upregulation of M1 inflammatory genes (NOS2, TNF-α, IL-1β, and IL-12) and a downregulation of M2 marker genes (ARG-1, IL10, and TGF-β) compared to cells treated with M.RGD@Cr-CTS-siNC NPs (Fig. 5C). Western blot analysis revealed no significant changes in ARG-1 and NOS2 protein levels in M0 BMDMs with or without M.RGD@Cr-CTS-siYTHDF1 treatment. In contrast, in M2-type BMDMs, ARG-1 was downregulated, and NOS2 was upregulated following treatment with M.RGD@Cr-CTS-siYTHDF1 NPs compared to M.RGD@Cr-CTS-siNC NPs (Fig. 5D). These findings align with previous studies investigating the involvement of YTHDF1 in macrophage polarization and the broader implications of m6A RNA methylation in immune regulation. Previous research has shown that YTHDF1, as an m6A reader, regulates important inflammatory genes (such as IFN-r, IL12, IL10, etc.), thereby influencing macrophage response to diverse stimuli and playing a critical role in macrophage function and polarization [30]. Future investigations should aim to uncover the precise mechanisms through which YTHDF1 governs macrophage polarization. Collectively, these results indicate that YTHDF1 depletion in M2-type macrophages promotes the expression of M1-type macrophage genes and proteins.

### Molecular mechanism of anti-tumor macrophage phenotype by YTHDF1

**Fig. 6:**
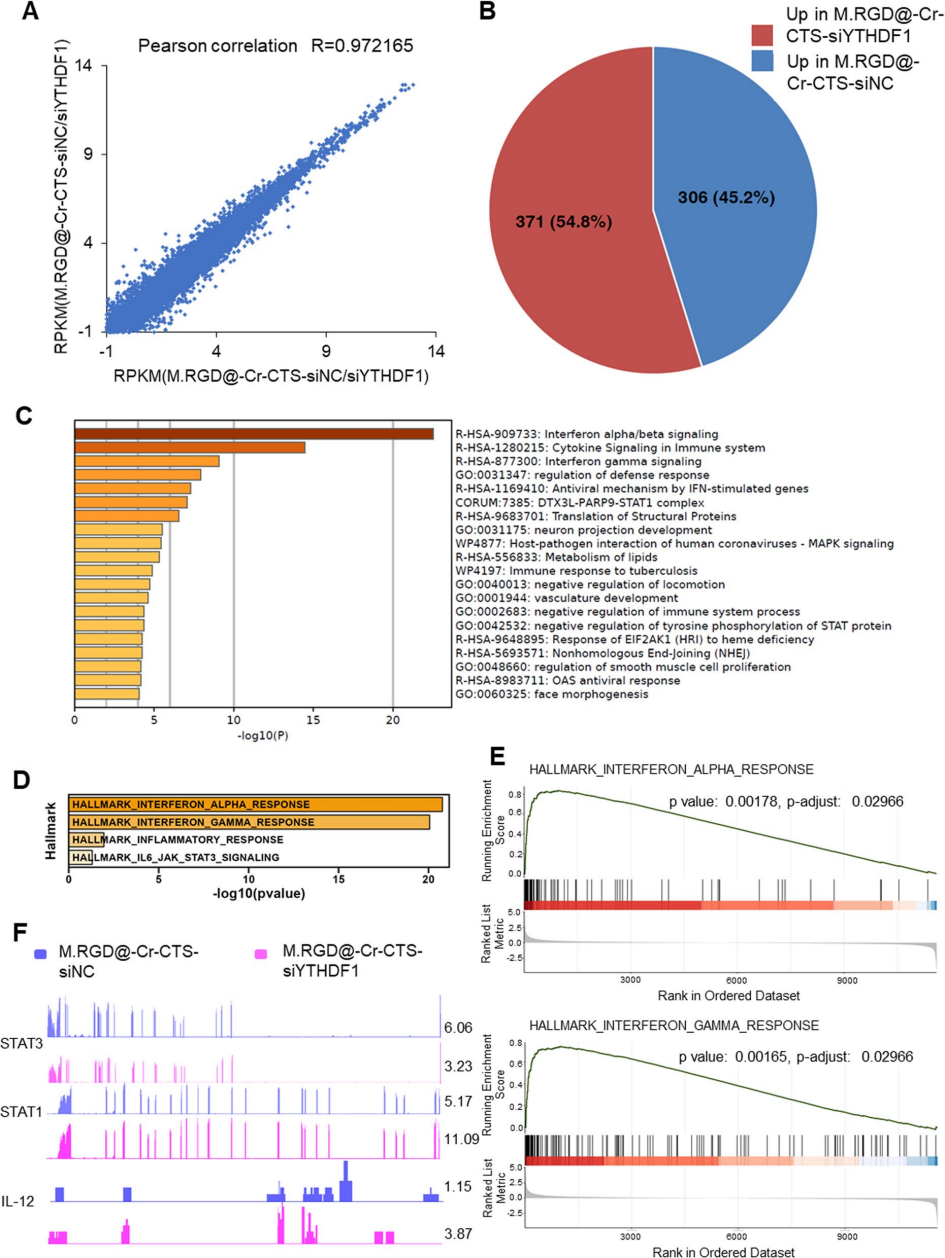

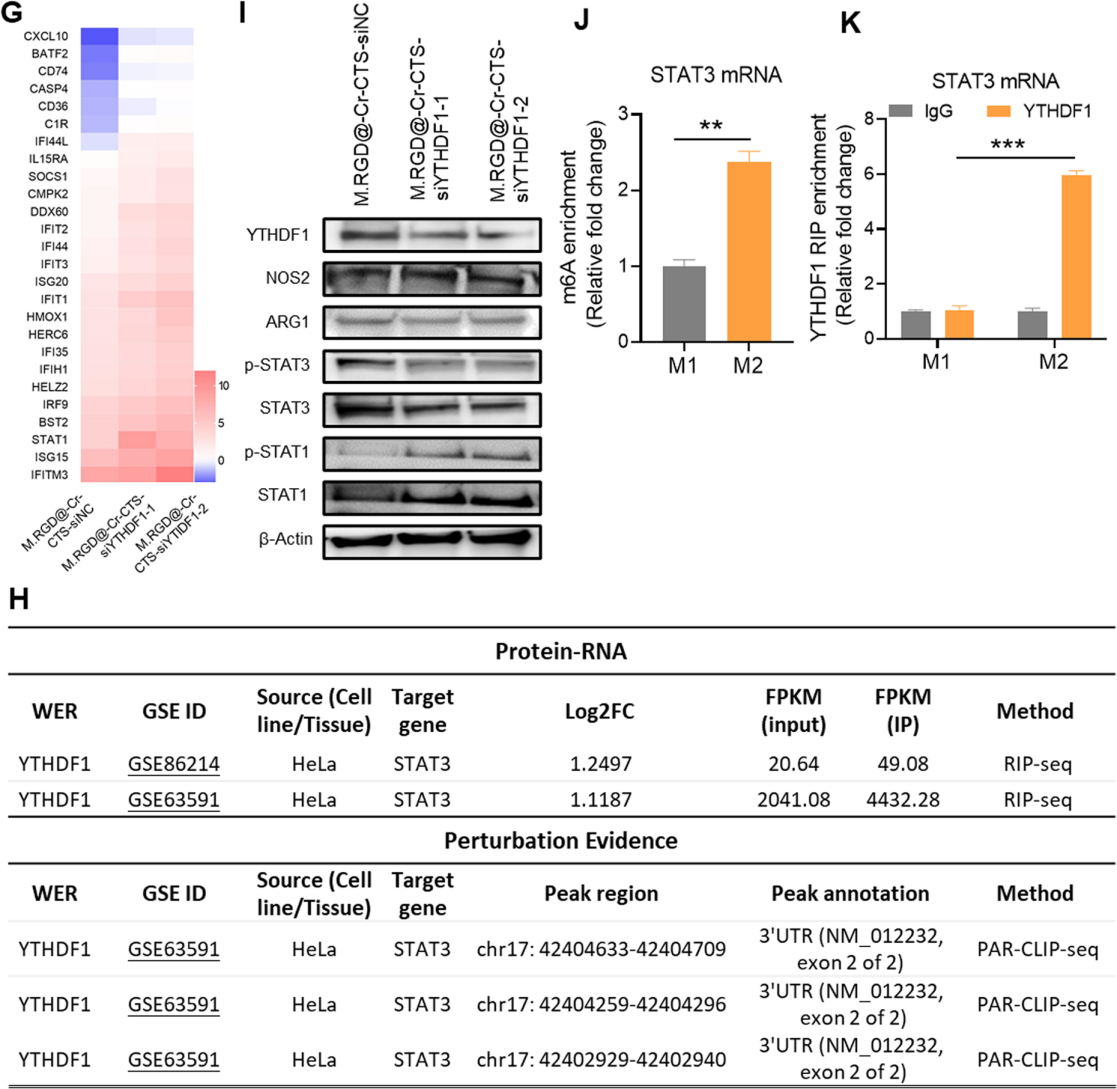
Identification of potential targets by YTHDF1 depletion via M.RGD@Cr-CTS-siYTHDF1 NPs treatment in THP-1 cells. **A** Pearson correlation analysis of RNA-seq results following YTHDF1 knockdown using two M.RGD@Cr-CTS-siYTHDF1 NPs treated THP-1 cells. **B** Pie chart of differentially expressed genes from RNA-seq results. **C** Ingenuity Pathway Analysis of upregulated genes. **D** Hallmark gene set enrichment analysis. **E** Gene Set Enrichment Analysis (GSEA) of gene set enrichment. **F** Genome browser views of RNA-seq for IL12, STAT3, and STAT1 as described in (**A**). **G** Heatmap of qPCR detection of inflammation-related gene expression following YTHDF1 knockdown in THP-1 cells induced to M2-type macrophages. **H** Prediction of the association between YTHDF1 and STAT3 binding and perturbation using the m6A2Target tool, and analysis of potential m6A sites on STAT3 mRNA using the m6Avar tool. **I** Western blot analysis of the expression of YTHDF1, STAT1, P-STAT1, STAT3, P-STAT3, NOS2, ARG-1 and β-ACTIN proteins after YTHDF1 knockdown in two M.RGD@Cr-CTS-siYTHDF1 NPs treated THP-1 cells. **J** qPCR analysis of STAT3 mRNA following m6A enrichment in THP-1 cells induced to M1 and M2-type macrophages. **K** RIP-qPCR analysis of the interaction between STAT3 mRNA and YTHDF1 protein in THP-1 derived M1 and M2-type macrophages. Data are presented as the means ± SD (n = 3), **p < 0.01, ***p < 0.01

To investigate the molecular mechanisms by which YTHDF1 modulates the anti-tumor properties of macrophages, we performed transcriptome sequencing on THP-1 cells with YTHDF1 knockdown. This was achieved using two distinct siRNAs (M.RGD@Cr-CTS-siYTHDF1-1 and M.RGD@Cr-CTS-siYTHDF1-2) to induce an M2-type macrophage phenotype. A high Pearson correlation coefficient of 0.97 between the two siRNA datasets indicated a strong consistency in YTHDF1 knockdown efficacy (Fig. 6A). Differential expression analysis identified 677 significantly altered genes, including STAT1 (Log2FC > 1.5 & p-value < 0.05: 371 upregulated and 306 downregulated genes) (Fig. 6B). Ingenuity Pathway Analysis (IPA) revealed that YTHDF1 knockdown in THP-1 pro-tumor macrophages activates the interferon (IFN) signaling pathway, IFN-regulated factors, and immune cell factor signaling (Fig. 6C). Gene Set Enrichment Analysis (GSEA) indicated an enrichment of inflammatory response genes in YTHDF1 knockdown anti-tumor macrophages, encompassing genes involved in IFN-γ signal transduction and functional genes such as upregulated STAT1, TNF-α, IL-12, JAK2, and IRF9, alongside downregulated STAT3 (Fig. 6E, F, Supplementary Fig. 6). Quantitative PCR (qPCR) validation confirmed significant upregulation of inflammatory response-related genes in YTHDF1 knockdown M2-type macrophages compared to THP-1-induced M2 macrophages (Fig. 6G). Prior studies have shown that STAT3 signaling antagonizes STAT1 expression, thereby inhibiting the IFN signaling pathway [31, 32].

Utilizing the m6A2Target tool, we predicted an interaction between YTHDF1 and STAT3 binding and modification. Analysis of potential m6A sites on STAT3 mRNA using m6Avar identified a plausible m6A site in exon 2 of the STAT3 transcript (Fig. 6H). YTHDF1 has been reported to regulate the methylation of STAT3 mRNA, leading to reduced levels of STAT3 and phosphorylated STAT3 protein [33, 34]. YTHDF1 knockdown resulted in decreased STAT3 expression and phosphorylation, along with elevated STAT1 expression and increased STAT1 phosphorylation (Fig. 6I). These changes suggest a promotion of anti-tumor macrophage polarization through the modulation of the STAT3-STAT1 balance in THP-1 cells. Previous research has established distinct roles for STAT3 and STAT1 in macrophage polarization. STAT3 promotes an M2 phenotype, often associated with tumor progression and immune suppression, while STAT1 is associated with the M1 phenotype, characterized by pro-inflammatory and anti-tumor activities. Xu et al. demonstrated that the m6A methylation machinery, including METTL3, facilitates M1 macrophage polarization by promoting the methylation and translation of STAT1 mRNA. The current study found that YTHDF1 knockdown decreases STAT3 phosphorylation and increases STAT1 phosphorylation, aligning with these roles [35]. Reduction of STAT3 through pharmacological or genetic methods in many systems enhances STAT1 activation. This is because both STAT1 and STAT3 bind to the same phosphorylated tyrosine residues in signaling proteins such as gp130, leading to increased phosphorylation of STAT1 protein due to reduced competition at these sites [36]. MeRIP-qPCR analysis showed that STAT3 mRNA has significantly higher m6A enrichment levels in M2 macrophages compared to M1, indicating the regulatory role of YTHDF1 in the methylation of STAT3 mRNA and its impact on STAT3 protein translation (Fig. 6J), consistent with the findings of Ito-Kureha et al. [37]. These results promote a shift in TAMs toward an M1 phenotype. RIP-qPCR further confirmed the direct binding of YTHDF1 to STAT3 mRNA in M2 macrophages compared to M1 macrophages (Fig. 6K). Therefore, our findings demonstrate that YTHDF1 regulates the methylation of STAT3 mRNA to suppress its expression, while simultaneously upregulating STAT1 expression. This dual action promotes a phenotypic shift towards the M1 macrophage phenotype.

### In vivo anti-tumor immune responses

**Fig. 7:**
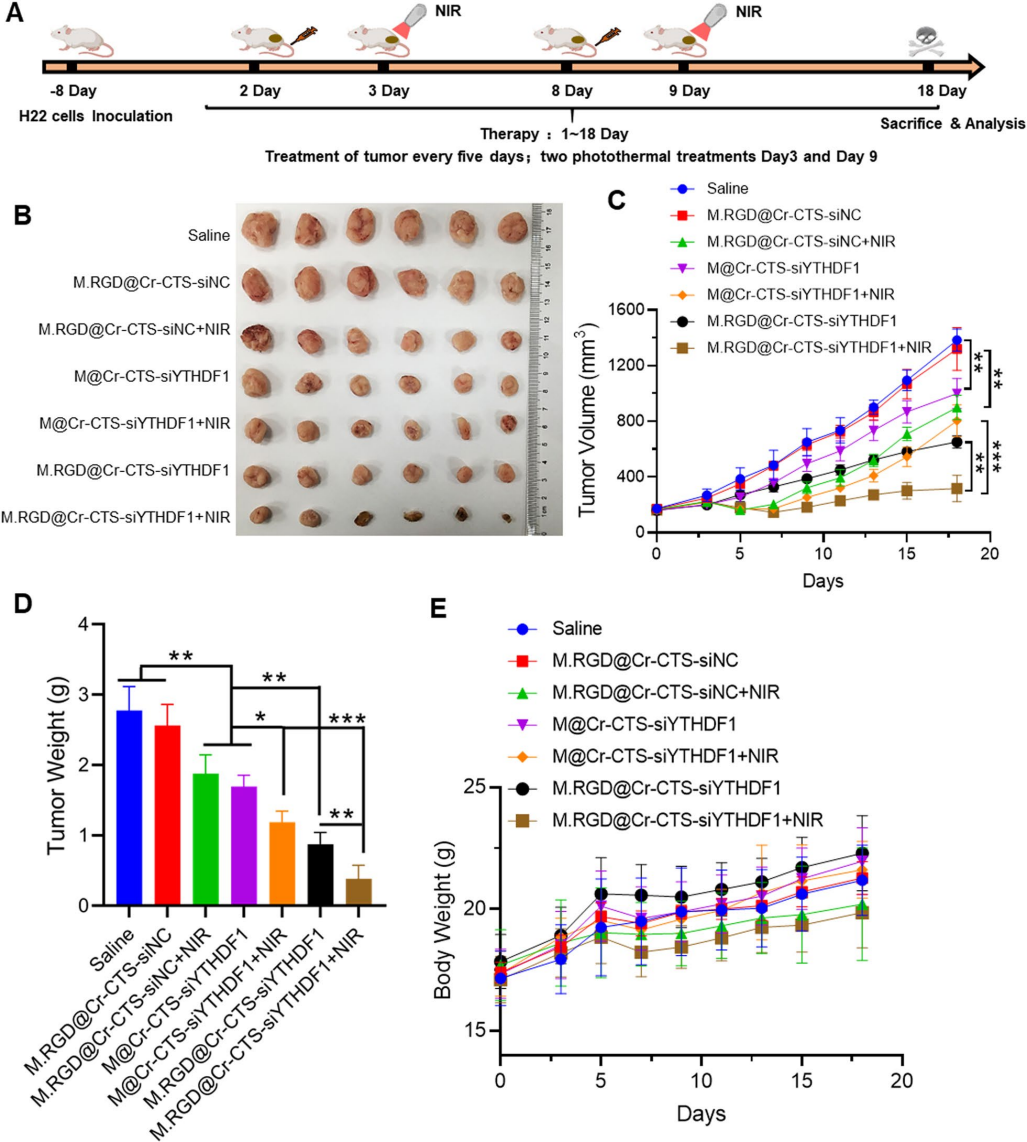
M.RGD@Cr-CTS-siYTHDF1 effectively inhibits subcutaneous hepatocellular carcinoma in mice. **A** Schematic representation of the treatment process using M.RGD@Cr-CTS-siYTHDF1 NPs in a mouse model of subcutaneous hepatocellular carcinoma. **B** Picture of the tumor after 18 days of treatment. **C** Tumor volume change curve of subcutaneous hepatocellular carcinoma in mice during various treatment regimens. **D** Tumor weight graph of subcutaneous hepatocellular carcinoma in mice following various treatments. **E** Curve showing changes in body weight over time after different treatments. Results are expressed as the mean ± standard deviation (n = 6). *, p < 0.05; **, p < 0.01; ***, p < 0.001

Immune checkpoint inhibitor (ICI) therapy faces challenges in treating solid tumors due to the complexity of the TME. A novel strategy employing laser photothermal effects to disrupt the TME has shown potential as an anti-tumor therapy [38]. We evaluated the in vivo anti-tumor efficacy of M.RGD@Cr-CTS-siYTHDF1 NPs in a mouse hepatic tumor model using H22 cells. When tumors reached approximately 100 mm^3^, mice were divided into seven groups: saline, M.RGD@Cr-CTS-siNC, M.RGD@Cr-CTS-siNC + NIR, M@Cr-CTS-siYTHDF1, M@Cr-CTS-siYTHDF1 + NIR, M.RGD@Cr-CTS-siYTHDF1, and M.RGD@Cr-CTS-siYTHDF1 + NIR. Each group received intravenous injections of the chromium nanoparticle formulation on day 2 and underwent near-infrared (NIR)-induced PTT at 24 h post-injection (1 W/cm^2^, 8 min). This treatment cycle was repeated after 5 days. On day 18, the mice were euthanized, and tumor volumes were analyzed (Fig. 7A). Tumor growth rate and mouse body weight were measured to assess the impact of different treatments. Figure 7B–D illustrates the significant inhibitory effect on tumor growth observed in both the M@Cr-CTS-siYTHDF1 and M.RGD@Cr-CTS-siYTHDF1 treatment groups, with inhibition rates of 53.1% and 51.4%, respectively, compared to the M.RGD@Cr-CTS-siNC group. These findings support previous studies highlighting the consistent impact of YTHDF1 siRNA on tumor growth [39, 40]. Additionally, combined photothermal therapy demonstrated a significant inhibition of tumor growth compared to the M.RGD@Cr-CTS-siNC and M.RGD@Cr-CTS-siYTHDF1 groups, with inhibition rates of 28.7% and 32.1%, respectively. Notably, mice treated with physiological saline and M.RGD@Cr-CTS-siNC exhibited faster tumor growth rates than the other treatment groups. Remarkably, the dual-targeted group M.RGD@Cr-CTS-siYTHDF1 + NIR, which combined photothermal and gene therapies, displayed the most substantial inhibitory effect on tumor growth, achieving a remarkable 78.2% reduction. This outcome surpassed the efficacy of single siRNA and photothermal therapy groups, highlighting the synergistic effect of Cr NPs’ PTT and YTHDF1 knockdown. The therapeutic potential of integrating PTT with targeted gene silencing has been previously reported by Zhang et al. and Zhai et al. [41, 42]. No significant changes in body weight were observed across all groups (Fig. 7E), indicating the treatment's safety. The combination therapy not only outperforms individual treatments but also emphasizes the significance of multi-modal therapeutic strategies in cancer treatment. Future research should focus on optimizing delivery systems and exploring the potential of such combinatory approaches in different cancer types to fully exploit their clinical benefits. These results suggest that M.RGD@Cr-CTS-siYTHDF1 NPs significantly impeded tumor progression.

### Immune modulation of M.RGD@Cr-CTS-siYTHDF1 NPs within TME

**Fig. 8:**
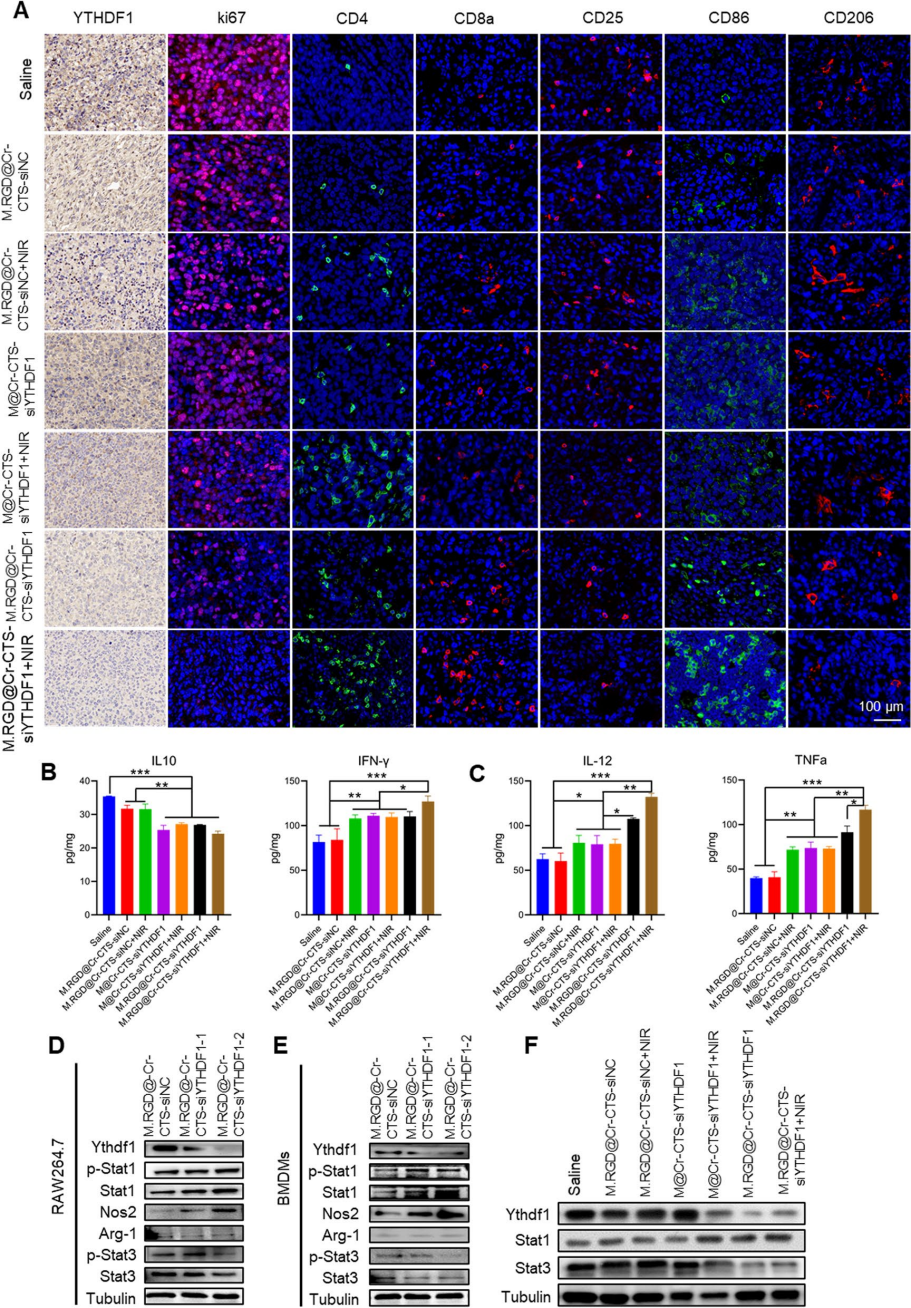
Immune regulation of M.RGD@Cr-CTS-siYTHDF1 in vivo. **A** Immunohistochemical staining for YTHDF1 and immunofluorescent staining for Ki67, CD4, CD8a, CD25, CD86, and CD206 in tumor tissues post-treatment. Scale bars = 100 µm. **B** Concentrations of intracellular cytokines IL10 and IFN-γ detected by ELISA. **C** Concentrations of serum cytokines IL-12 and TNFα detected by ELISA. Data are presented as mean ± S.D. (n = 3), *p < 0.05, **p < 0.01, ***p < 0.001 (non-repeated ANOVA followed by the SNK test). **D**, **E** Western blotting showing protein expression of YTHDF1, STAT1, P-STAT1, STAT3, P-STAT3, NOS2, ARG-1 and Tubulin in RAW264.7 (**D**) and HGC-27 (**E**) cells after treated with different M.RGD@Cr-CTS-siYTHDF1 NPs. **F** Western blot analysis of the expression of YTHDF1, STAT3, and Tubulin in hepatocellular carcinoma tissues following various treatments

We examined the potential anti-tumor mechanisms by assessing whether the combination of M.RGD@Cr-CTS-siYTHDF1 and PTT could effectively inhibit subcutaneous hepatocellular carcinoma growth in mice and enhance immune cell infiltration within the tumor. Immunohistochemical analysis showed a significant reduction in YTHDF1 gene expression in the siRNA gene therapy groups (M@Cr-CTS-siYTHDF1 and M.RGD@Cr-CTS-siYTHDF1), indicating the nanoparticles' ability to target tumor cells for YTHDF1 knockdown (Fig. 8A). This knockdown correlates with reduced tumor cell proliferation, as evidenced by decreased Ki67 + staining, and increased tumor cell necrosis, particularly in groups treated with PTT (Fig. 8A). These findings are consistent with previous research indicating that YTHDF1 plays a crucial role in promoting tumor growth and proliferation through its regulation of m6A-modified mRNA translation [43]. T cell infiltration is essential for cancer immunotherapy [44]. Importantly, the combination treatment led to a marked increase in CD8 + and CD4 + T cell infiltration, with the M.RGD@Cr-CTS-siYTHDF1 + NIR group showing the most pronounced immune cell presence (Fig. 8A). This enhanced infiltration is critical for effective cancer immunotherapy, as robust T cell activity is associated with better anti-tumor responses [18, 45]. Furthermore, the shift towards a pro-inflammatory tumor microenvironment was evident from the increased presence of M1-type TAMs (CD86) and decreased M2-type TAMs (CD206) and regulatory T cells (Tregs, CD25) in the combination treatment group (Fig. 8A). This shift is supported by elevated levels of pro-inflammatory cytokines (IFN-γ, IL-12, and TNF-α) and reduced levels of the anti-inflammatory cytokine IL-10 in mice serum intracellular cytokines, aligning with previous studies that have shown similar cytokine expression patterns following YTHDF1 deletion (Fig. 8B, C) [12, 26, 46, 47]. Overall, the synergistic effect of YTHDF1 knockdown and PTT not only directly suppresses tumor cell proliferation but also modulates the immune microenvironment to favor anti-tumor immunity.

The effects of M.RGD@Cr-CTS-siYTHDF1 NPs on STAT3 and STAT1 were further investigated in RAW264.7 and murine bone marrow-derived macrophages (BMDMs). Western blot analysis showed that treatment with M.RGD@Cr-CTS-siYTHDF1 NPs significantly reduced Arg-1 and STAT3 levels and their phosphorylation, while increasing Nos2 and STAT1 expression, indicate a shift towards an anti-tumor polarization of macrophages (Fig. 8D, E). Consistent with these findings, Western blot analysis of tumor tissue revealed that M.RGD@Cr-CTS-siYTHDF1 treatment significantly knocked down YTHDF1, decreased STAT3, and enhanced STAT1 expression (Fig. 8F). These results were consistent with previous reports showing that YTHDF1 gene deletion reduced STAT3 protein levels and phosphorylation, disrupted the STAT3-STAT1 balance in macrophages, promoted STAT1 expression and phosphorylation, and induced an anti-tumor polarization of macrophages [36, 48]. Thus, our study provides valuable insights into the molecular mechanisms underlying the combination therapy and highlights its potential to enhance the efficacy of existing immunotherapies. Future studies should focus on exploring the clinical applicability of this combination therapy and conducting further investigations to validate its effectiveness in improving patient outcomes.

## Conclusions

In summary, we have developed a novel dual-targeting system, M.RGD@Cr-CTS-siYTHDF1 NPs, which utilize RGD to target tumor cells and Cr nano-laser photothermal technology to disrupt the tumor microenvironment and employ mannose to target TAMs for effective delivery of siYTHDF1. The technology of light control and gene silencing is harnessed to establish a therapeutic gene regulation system with the m6A-modified reading gene YTHDF1 as the core. Mechanistically, this system decreases STAT3 protein expression, disrupts the STAT3-STAT1 balance in TAMs, enhances STAT1 expression, and induces TAM polarization towards the M1 phenotype. These changes result in a significant reduction in TAMs, TME remodeling, reversal of tumor immunosuppression, and ultimately inhibition of liver tumor growth. Moreover, the study has several limitations that should be considered. Firstly, our evaluation of targeted delivery and treatment was limited to a mouse model of liver cancer, and further investigation is required to explore the therapeutic effects of M.RGD@Cr-CTS-siYTHDF1 NPs on mouse metastatic liver cancer. Secondly, although M.RGD@Cr-CTS-siYTHDF1 NPs demonstrated effective targeting and delivery of siRNA to tumors, their transient transfection efficiency may hinder their clinical applications. To address this issue, advanced technologies such as transposons and CRISPR-based genome editing systems could be explored to prolong siRNA expression and ensure sustained stability. Thirdly, it is important to note that laser photothermal transmission has limited penetration depth, posing challenges for treating deep-seated tumors within the body. This study emphasizes the significance of multifunctional nanoparticles for TAM targeting, enhances m6A modulator-targeted anti-tumor therapeutic strategies, and broadens the therapeutic options for malignant tumors, such as hepatocellular carcinoma.

### Supplementary Information

Additional file 1.

Additional file 2.

## Data Availability

The authors declare that all data supporting the results in this study are available in the paper and Supplementary Materials. Source data are available from the corresponding authors upon reasonable request.

## Abbreviations

AFM: Atomic force microscopy
H&E: Hematoxylin and eosin
NIR: Near-infrared light
NPs: Nanoparticles
PTT: Photothermal therapy
TEM: Transmission electron microscopy
TME: Tumor microenvironment
Man-COOH (M.): Carboxymethyl mannose
CTS: Chitosan
BMDMs: Bone marrow-derived macrophages
ELISA: Enzyme-linked immunoassay
