# Supplementary material for: Photosensitive and dual-targeted chromium nanoparticle delivering small interfering RNA YTHDF1 for molecular-targeted immunotherapy in liver cancer
Source: J Nanobiotechnology. 2024 Jun 19;22:348. doi: 10.1186/s12951-024-02612-3 (PMC11188166; doi:10.1186/s12951-024-02612-3)
Supplement: Supplementary file 1 — Additional file 1. [file 12951_2024_2612_MOESM1_ESM.docx]

**Photosensitive and Dual-targeted Chromium Nanoparticle Delivering Small Interfering RNA YTHDF1 for Molecular-Targeted Immunotherapy in Liver Cancer**

Shang Chen^1,2^, Yan He^1,3^, Xin Huang^1,4^, Yao Shen^1^, Qingshuang Zou^5^, Gun Yang^1,3^, Li Fu^6,*^, Quan Liu^1,*^, and Dixian Luo^1,*^

^1^Department of Laboratory Medicine, Huazhong University of Science and Technology Union Shenzhen Hospital (Nanshan Hospital), Shenzhen University, Shenzhen, 518052, P. R. China.

^2^Guangdong Key Laboratory for Biomedical Measurements and Ultrasound Imaging, National-Regional Key Technology Engineering Laboratory for Medical Ultrasound, School of Biomedical Engineering, Shenzhen University Medical School, Shenzhen, 518060, P. R. China.

^3^Institute of Pharmacy and Pharmacology, School of Pharmaceutical Science, Hengyang Medical School, University of South China, Hengyang, 421001, P. R. China.

^4^Department of Thoracic Surgery, First Affiliated Hospital of Anhui Medical University, Hefei 230032, P. R. China.

^5^Department of Chemistry, The Chinese University of Hong Kong, Shatin, N.T., Hong Kong, PR China

^6^Guangdong Provincial Key Laboratory of Regional Immunity and Diseases, Department of Pharmacology and International Cancer Center, Shenzhen University Health Science Center, Shenzhen, 518055, P. R. China.

^7^National Forestry and Grassland Administration Key Laboratory of Plant Fiber Functional Materials, College of Materials Engineering, Fujian Agriculture and Forestry University, Fuzhou 350002, China

*Corresponding e-mail: liu_quan2020@163.com (Q.L.); gracelfu@szu.edu.cn (F.L.); luodixian_2@163.com (D.L.)

**Supplementary Figures**


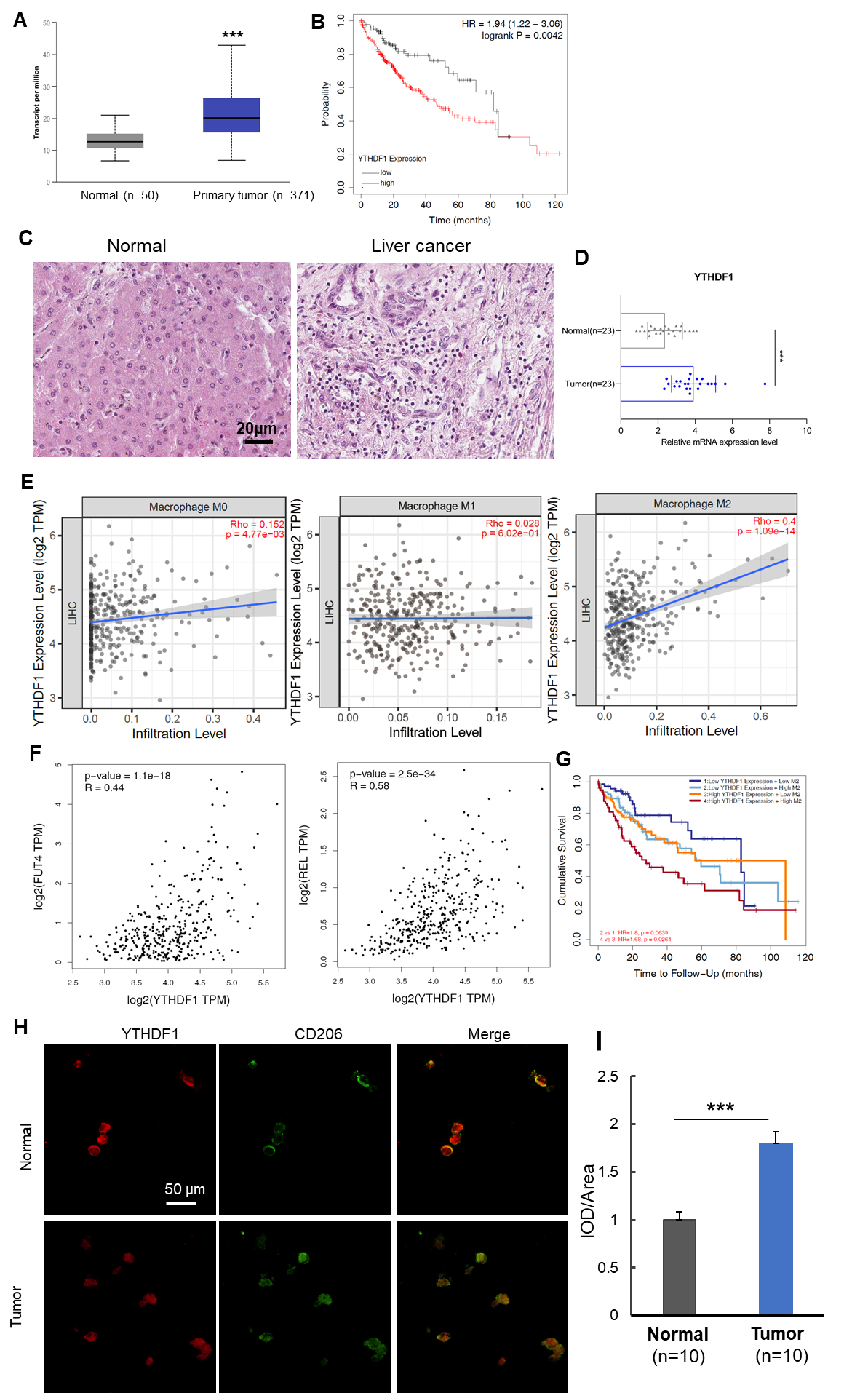


**Supplementary Figure 1.** YTHDF1 Expression Patterns in Liver Tumors and Infiltrating Cells within the Tumor Microenvironment. (A) Examination of YTHDF1 expression in hepatocellular carcinoma using data from The Cancer Genome Atlas (TCGA) database. (B) Kaplan-Meier survival curves based on YTHDF1 expression for patient prognosis. (C) Immunohistochemical analysis of 23 pairs of clinical hepatocellular carcinoma samples. (D) Analysis of YTHDF1 mRNA expression in clinical hepatocellular carcinoma tissue samples. (E) Correlation analysis between YTHDF1 expression levels and infiltration levels of different macrophage types (M0, M1, M2) in hepatocellular carcinoma. Data is presented with a fitted line at a 95% confidence level, analyzed using a linear model. (F) Correlation between myeloid-related genes REL and FUT4 expression levels and YTHDF1 gene expression in immune-infiltrating cells of hepatocellular carcinoma from the TIMER 2.0 database. (G) Kaplan-Meier survival curve analysis based on YTHDF1 expression levels and M2-type macrophage infiltration levels for patient prognosis. (H) Co-localization distribution of M2-type tumor-associated macrophages (TAMs, CD206 in red) and YTHDF1 (in green) in hepatocellular carcinoma tissue samples. (I) Statistical analysis of M2-type TAMs and YTHDF1 co-localization in 10 pairs of liver cancer samples ***p<0.001.

**Supplementary Figure 2.** Characterization of M.RGD@Cr-CTS-siYTHDF1 NPs UV-Vis-NIR absorption spectra (λ= 808 nm) of dispersions with varying concentrations of Cr NP, M.RGD@Cr-siYTHDF1, M.@Cr-CTS-siYTHDF1, and M.RGD@Cr-CTS-siYTHDF1.


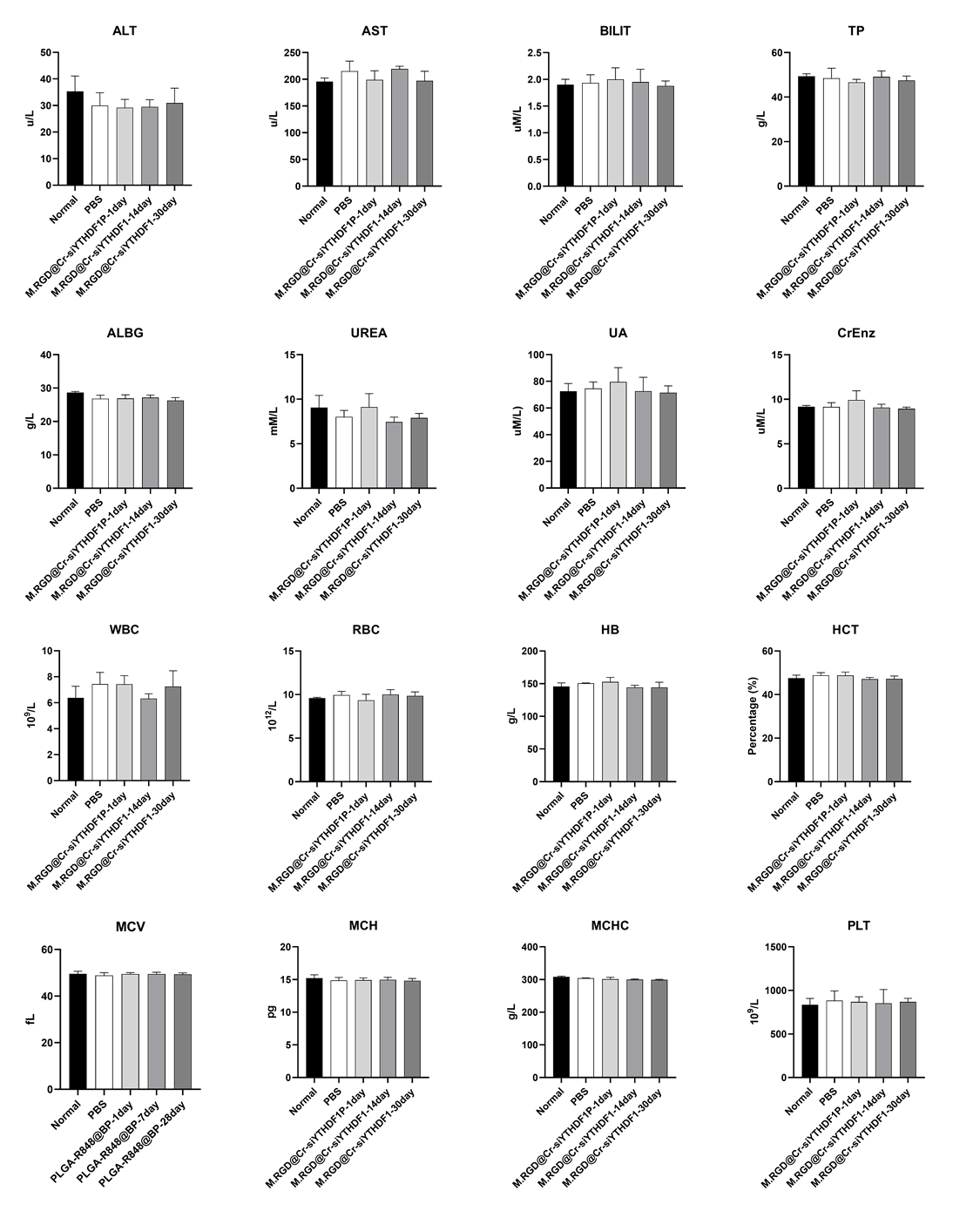


**Supplementary Figure 3. Evaluation of blood profile, urine profile, liver function, and kidney function.** Hematological analysis of the mice injected with Saline or M.RGD@Cr-CTS-siYTHDF1 at Day 1, Day 14, and Day 30. WBC, white blood cells; RBC, red blood cell; HB, Hemoglobin; HCT, Hematocrit; MCV, mean corpuscular volume; MCH, mean corpuscular hemoglobin; MCHC, mean corpusular hemoglobin concerntration; PLT, platelet count; ALT, aspartate transaminase; AST, aspartate aminotransferase; BILIT, bilirubin; TP, total protein; ALBG, albumin; UREA, urea; UA, uric acid; CrEnz, creatinine enzyme.


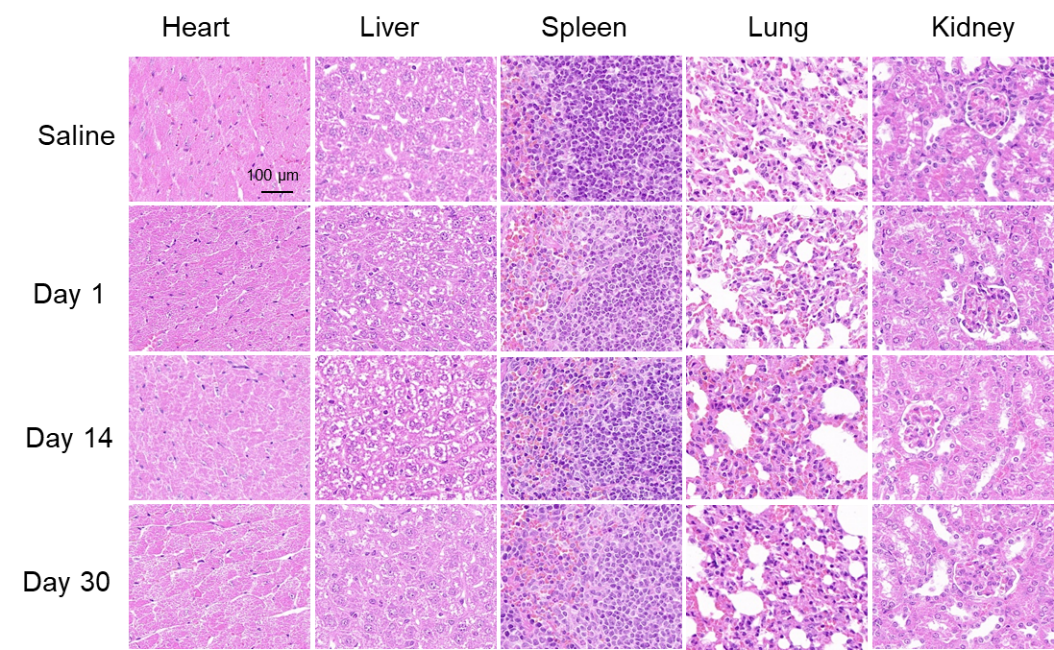


**Supplementary Figure 4.** Pathological toxicity analysis of main organs. H&E analysis of the mice injected with Saline or M.RGD@Cr-CTS-siYTHDF1 at Day 1, Day 14, and Day 30. H&E staining images of vital organs such as the heart, liver, spleen, lung, and kidney from treated mice with saline and M.RGD@Cr-CTS-siYTHDF1. Scale bar = 100 μm.


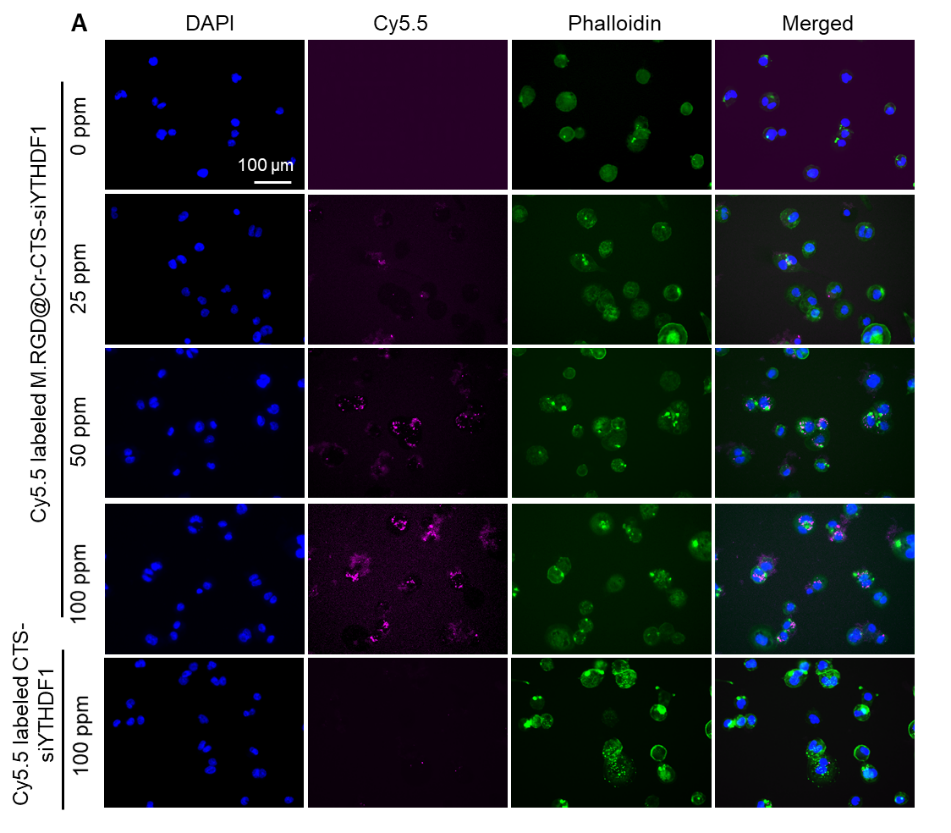


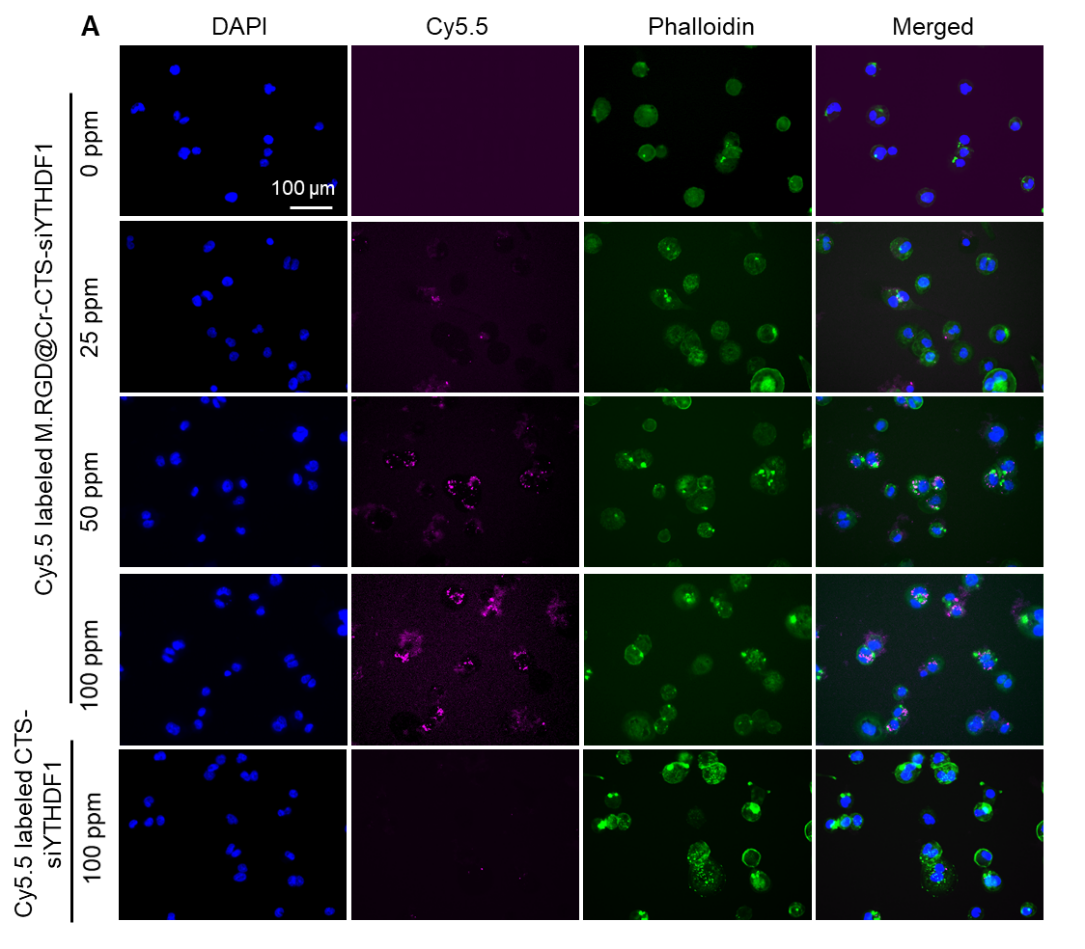


**Supplementary Figure 5.** A, B. Confocal microscopy visualization of the uptake of Cy5.5-labeled M.RGD@Cr-CTS-siYTHDF1 NPs and Cy5.5-labeled CTS-siYTHDF1 by Hepa1-6 (A) and RAW264.7 cells (B) with different concentrations.


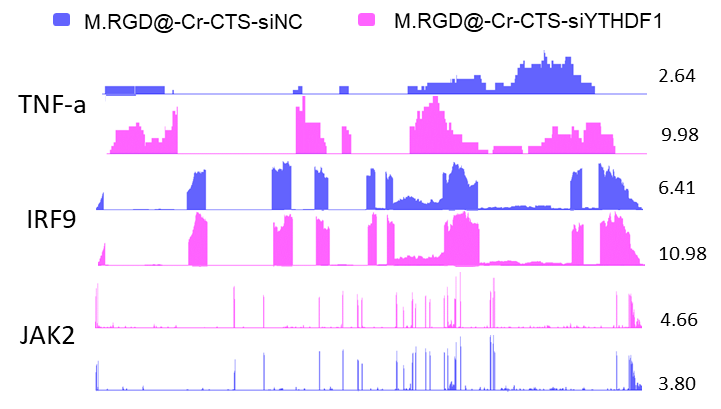


**Supplementary Figure 6.** Distribution of PLGA@BP-R848 in tumor tissue. Genome browser views of RNA-seq for TNF-a, JAK2, and IRF9.
